# Mitochondria‐Targeted Multifunctional Nanoparticles Combine Cuproptosis and Programmed Cell Death‐1 Downregulation for Cancer Immunotherapy

**DOI:** 10.1002/advs.202403520

**Published:** 2024-07-16

**Authors:** Youyou Li, Jing Liu, Ralph R. Weichselbaum, Wenbin Lin

**Affiliations:** ^1^ Department of Chemistry University of Chicago 929 East 57th Street Chicago IL 60637 USA; ^2^ Department of Radiation and Cellular Oncology and Ludwig Center for Metastasis Research University of Chicago 5758 South Maryland Avenue Chicago IL 60637 USA

**Keywords:** cancer immunotherapy, cuproptosis, mitochondria‐targeting, nanomedicine, PD‐L1 downregulation

## Abstract

The combination of cuproptosis and immune checkpoint inhibition has shown promise in treating malignant tumors. However, it remains a challenge to deliver copper ions and immune checkpoint inhibitors efficiently and simultaneously to tumors. Herein, a mitochondria‐targeted nanoscale coordination polymer particle, Cu/TI, comprising Cu(II), and a triphenylphosphonium conjugate of 5‐carboxy‐8‐hydroxyquinoline (TI), for effective cuproptosis induction and programmed cell death‐1 (PD‐L1) downregulation is reported. Upon systemic administration, Cu/TI efficiently accumulates in tumor tissues to induce immunogenic cancer cell death and reduce PD‐L1 expression. Consequently, Cu/TI promotes the intratumoral infiltration and activation of cytotoxic T lymphocytes to greatly inhibit tumor progression of colorectal carcinoma and triple‐negative breast cancer in mouse models without causing obvious side effects.

## Graphical abstract

Mitochondria‐targeted core–shell nanoparticle Cu/TI for simultaneous cuproptosis induction and PD‐L1 downregulation is reported. Cu/TI exhibits prolonged blood circulation and high tumor uptake to induce immunogenic cancer cell death and reduce PD‐L1 expression in vivo, thereby effectively enhancing the infiltration and activation of cytotoxic T lymphocytes to elicit potent antitumor effects in mouse models without causing obvious side effects.

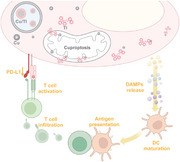

## 1 Introduction

The discovery of novel pathways of cell death holds immense promise in killing cancer cells that are resistant to traditional apoptosis and necroptosis mechanisms.^[^ ^1^ ^]^ The recent discovery of cuproptosis has spurred interest in exploiting this novel variant of regulated cell death for cancer therapy.^[^ ^2^ ^]^ As cuproptosis depends on a high concentration of mitochondrial copper (Cu) ^[^ ^2^, ^3^ ^]^ and yet intracellular labile Cu concentrations are extremely low,^[^ ^4^ ^]^ the triggering of cuproptosis critically depends on Cu ionophores for transporting extracellular Cu into cellular mitochondria.^[^ ^4^, ^5^ ^]^ Although many Cu ionophores, such as disulfiram, 8‐hydroxyquinoline, pyrithione, and elesclomol have been developed, they are ineffective cancer therapeutics due to their lack of tumor specificity, rapid elimination, and active metabolism in vivo.^[^ ^6^ ^]^ Consequently, the efficient transport of Cu into the mitochondria of cancer cells in vivo presents a bottleneck for harnessing cuproptosis as an effective anticancer treatment. We hypothesized that nanoparticles may provide a potential solution to selective delivery of Cu into the mitochondria of cancer cells for triggering cuproptosis.^[^ ^7^ ^]^

The use of Cu ionophores to transport Cu into tumor cells has been shown to upregulate programmed cell death‐1 (PD‐L1) expression.^[^ ^8^ ^]^ Although anti‐PD‐L1 antibodies (αPD‐L1) have been combined with Cu ionophore treatment to enhance cancer treatment efficacy,^[^ ^9^ ^]^ reducing the natural expression of PD‐L1 in tumors offers an interesting alternative to the blockade of the PD‐L1 checkpoint using monoclonal antibodies.^[^ ^10^ ^]^ By targeting specific biochemical pathways that are upregulated in cancer cells while sparing normal tissues, this approach holds the promise of circumventing immune‐related adverse events.^[^ ^11^ ^]^ Direct downregulation of PD‐L1 expression in tumors can also reduce membrane‐bound PD‐L1 to elicit more effective activation of cytotoxic T cells.^[^ ^12^ ^]^ Consequently, there is a strong interest in developing small molecule inhibitors to target the PD‐L1 axis.^[^ ^13^ ^]^ Among them, the JMJD1A inhibitor 5‐carboxy‐8‐hydroxyquinoline (IOX1) has recently been shown to effectively inhibit tumor PD‐L1 expression.^[^ ^8^, ^14^ ^]^

**Figure 1:**
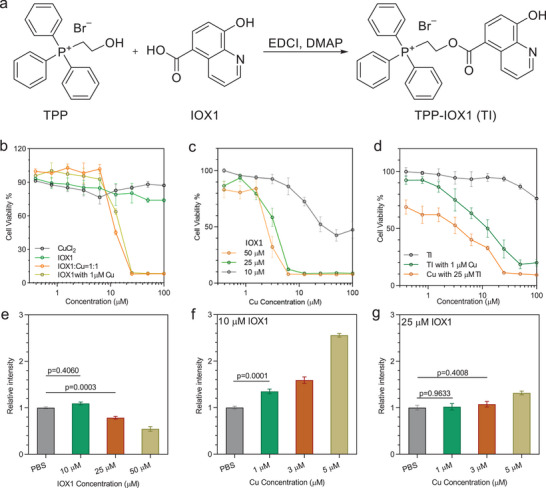
a) Synthesis of TPP‐IOX1 (TI). b–d) Cytotoxicity of Cu, IOX1, and TI under different conditions. e–g) PD‐L1 expression levels after different treatments. Data are represented as mean ± SD, *n* = 3 in (b–g). One‐way ANOVA with Tukey's correction for statistical significance.

Herein, we designed a novel core–shell nanoscale coordination polymer (NCP) nanoparticle, Cu/TI, with Cu(II) in the core and a triphenylphosphonium (TPP) conjugate of IOX1 (TI) (**Figure** **1**a) in the shell. Upon uptake of Cu/TI by cancer cells, TI not only acts as an ionophore to transport Cu(II) ions to mitochondria for cuproptosis induction, but also downregulates PD‐L1 to overcome immune suppression. Intravenous injection of Cu/TI released danger‐associated molecular patterns (DAMPs) and significantly downregulated PD‐L1 expression in tumors, thereby efficiently promoting the intratumoral infiltration and activation of cytotoxic T lymphocytes. As a result, Cu/TI significantly suppressed the growth of colorectal carcinoma and triple‐negative breast cancer (TNBC) in mouse models without inducing obvious side effects. This work highlights the potential of NCPs in the codelivery of multiple drugs to specific organelles for potent cancer treatment.

## 2 Results and Discussion

### 2.1 IOX1 Enhances Cu Cytotoxicity and Inhibits PD‐L1 Expression

8‐Hydroxyquinoline and its derivatives are reported to function as Cu ionophores to mediate cell death.^[^ ^6c^ ^]^ We tested the cytotoxicity of IOX1 in combination with Cu. Neither CuCl_2_ nor IOX1 induced significant death of 4T1 cancer cells at concentrations of up to 100 µm (Figure 1b; and Table S1, Supporting Information). However, the cytotoxicity of IOX1 significantly increased in the presence of 1 µm CuCl_2_ with a half‐maximal inhibitory concentration (IC_50_) of 14.36 ± 0.73 µm (Figure 1b; and Table S1, Supporting Information). Similarly, the Cu IC_50_ values decreased to 40.93 ± 10.22, 3.19 ± 0.87, and 2.28 ± 0.70 µm in the presence of 10, 25, and 50 µm IOX1, respectively (Figure 1c; and Table S2, Supporting Information). These results show that IOX1 enhances Cu cytotoxicity in a concentration‐dependent manner, likely due to its ability to transport Cu intracellularly to mediate cancer cell death. Notably, IOX1 did not enhance the cytotoxicity of iron and zinc (Figure S1, Supporting Information), which further supports the role of IOX1 as an ionophore for intracellular trafficking of Cu(II) ions.

IOX1 has been reported to downregulate PD‐L1 expression.^[^ ^8^, ^14^ ^]^ We evaluated the impact of IOX1 and Cu cotreatment on PD‐L1 expression in 4T1 cells by flow cytometry. In the absence of Cu, treatment with 10 µm IOX1 did not downregulate PD‐L1 expression but treatments with 25 and 50 µm IOX1 reduced PD‐L1 expression by 21.4% and 45.3%, respectively, from the PBS control (Figure 1e; and Figure S2, Supporting Information). Treatment of 4T1 cells with up to 5 µm Cu did not obviously increase PD‐L1 expression (Figure S3, Supporting Information). Cotreatment of 4T1 cells by 1, 3, and 5 µm Cu with 10 µm IOX1 increased PD‐L1 expression by 34.8%, 59.1%, and 155.7%, respectively, from the level of the phosphate‐buffered saline (PBS) control (Figure 1f; and Figure S2, Supporting Information). In contrast, cotreatment of 4T1 cells with 25 µm IOX1 reduced PD‐L1 expressions in 1 and 3 µm Cu groups to the levels of the PBS control, but was not able to completely abrogate PD‐L1 upregulation caused by 5 µm Cu treatment (Figure 1g; and Figure S2, Supporting Information). These results show that IOX1 not only enhances Cu cytotoxicity as an ionophore but also dose‐dependently inhibits PD‐L1 expression caused by Cu treatment.

### 2.2 TPP‐IOX1 Enhances Cu Accumulation in Mitochondria

To specifically deliver Cu to the mitochondria of cancer cells and induce cell cuproptosis, we conjugated IOX1 with the triphenylphosphonium (TPP) group that is known to target mitochondria.^[^ ^15^ ^]^ TPP‐IOX1 (TI) was synthesized by esterification between IOX1 and (2‐hydroxyethyl)triphenylphosphonium bromide in the presence of 1‐ethyl‐3‐(3‐dimethylaminopropyl)carbodiimide and 4‐dimethylaminopyridine and purified by silica gel column chromatography. TI was characterized by ^1^H and ^13^C NMR spectroscopy and high‐resolution mass spectrometry (Figures S4–S8, Supporting Information).

TI did not cause cell death at a concentration of 100 µm (Figure 1d; and Table S3, Supporting Information), and showed a comparable IC_50_ (11.75 ± 2.38 µm) to IOX1 in the presence of 1 µm Cu. The IC_50_ of Cu was 2.54 ± 0.70 µm in the presence of 25 µm TI, which is slightly lower than the Cu IC_50_ of 3.19 ± 0.87 in presence of 25 µm IOX1. The slightly enhanced cytotoxicity of the Cu and TI combination over the Cu and IOX1 combination is likely due to the mitochondrial‐targeting property of TI.

**Figure 2:**
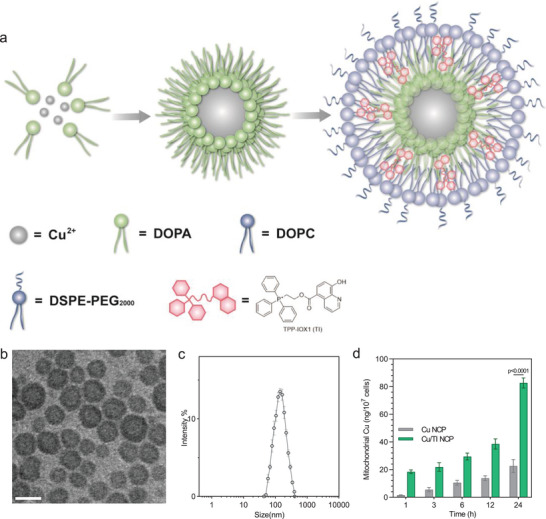
a) Schematic illustration of Cu/TI NCP. b) TEM image of Cu/TI NCP. Scale bar is 100 nm. c) Size distribution of Cu/TI NCP by DLS. d) Mitochondrial Cu amount by ICP‐MS. Data are represented as mean ± SD, *n* = 3 in (c,d). One‐way ANOVA with Tukey's correction for statistical significance.

To selectively deliver Cu and TI to tumors in vivo, we prepared the nanoscale coordination polymer (NCP) particle Cu/TI by incorporating Cu^2+^ ions in the Cu‐phosphate coordination polymer core and TI in the lipid shell (**Figure** **2**a). Cu/TI displayed a sphere morphology under TEM (Figure 2b) and exhibited a hydrodynamic size of 137.5 ± 2.4 nm (Figure 2c) with a polydispersity index (PDI) of 0.237 by dynamic light scattering. The zeta potential of Cu/TI was measured to be 1.3 ± 0.3 mV. Three different batches of Cu/TI showed consistent particle sizes, demonstrating reproducibility of Cu/TI nanoparticles (Figure S9, Supporting Information). Cu/TI displayed unaltered particle size after incubation in PBS for 1 week (Figure S9, Supporting Information).

We also synthesized the TPP‐pyro conjugate (Figures S10–S12, Supporting Information) as a fluorescent surrogate for TI to study the mitochondrial targeting ability of TI. TPP‐pyro and MitoTracker showed increased colocalization as the incubation time increased, with colocalization coefficients of 0.34, 0.76, and 0.89 after incubation for 1, 3, and 6 h, respectively (Figure S13, Supporting Information). This result suggests that TI can target mitochondria of cancer cells. We further determined the Cu amount in mitochondria by inductively coupled plasma‐mass spectrometry (ICP‐MS) after extracting the mitochondria from the treated cells. TI increased Cu accumulation in the mitochondria by 3.7‐fold over Cu‐NCP, a control particle with only Cu in the core (and without TI in the shell) (Figure 2d).

### 2.3 Cu/TI Causes Cell Cuproptosis

**Figure 3:**
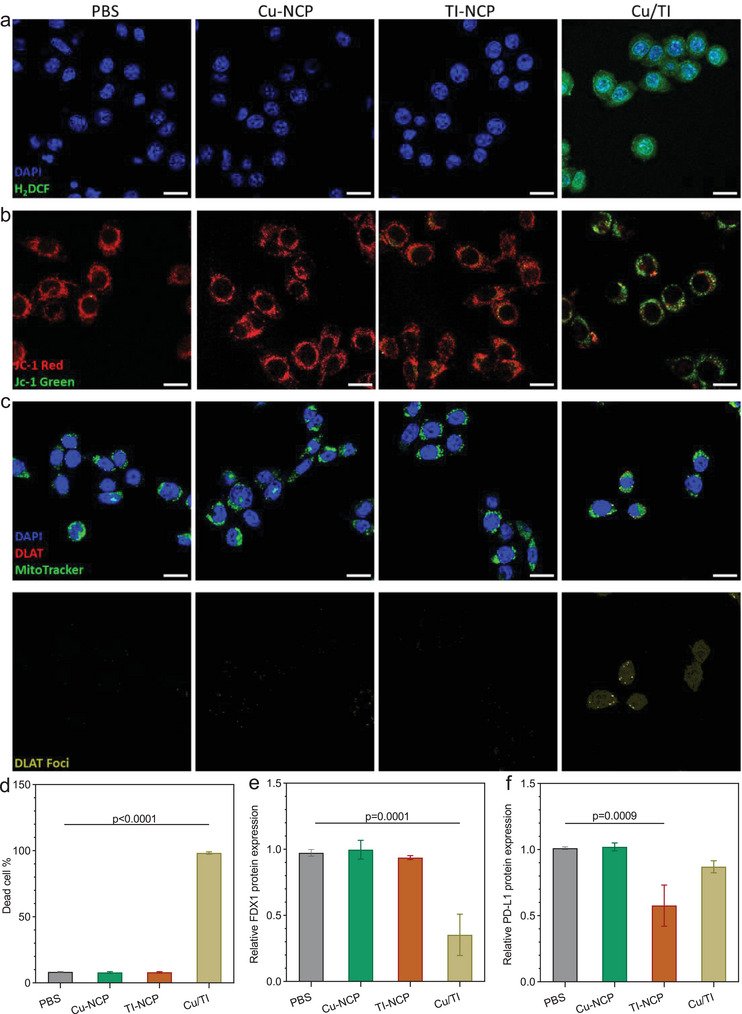
a) ROS generation, b) mitochondrial membrane potential change, and c) DLAT foci numbers of 4T1 cells after different treatments. Scale bars are 25 µm. d) Percentages of dead 4T1 cells after different treatments. Relative e) FDX1 and f) PD‐L1 protein expressions in 4T1 cells after different treatments by western blot. Data are represented as mean ± SD, *n* = 3 in (d–f). One‐way ANOVA with Tukey's correction for statistical significance.

Cu/TI particles showed much enhanced cytotoxicity in 4T1 cells compared to other groups at equivalent concentrations of 1 µm Cu and 25 µm TI. Cu/TI treatment led to 98.3% cell death while PBS, Cu‐NCP, and TI‐NCP (a control particle with only TI in the shell) treatments resulted in 8.3%,7.9%, and 8.0% cell death, respectively (**Figure** **3**d; and Figure S14, Supporting Information). Cu/TI treatment induced much higher reactive oxygen species (ROS) levels in 4T1 cells than other groups (Figure 3a), suggesting the synergy between Cu and TI to induce mitochondrial oxidative stress and cause cell death.

We also assessed mitochondrial membrane integrity by JC‐1 staining to probe the membrane potential change. The percentages of JC‐1 green, which correlate with abnormal mitochondrial membrane potentials, were 15.3% and 55.9% in TI‐NCP and Cu/TI treated cells, respectively (Figure 3b; and Figure S15, Supporting Information). In comparison, the other control groups did not show JC‐1 green signals.

We next determined if Cu/TI could mediate cuproptosis of 4T1 cells. Cuproptosis is characterized by an increased number of dihydrolipoyl transacetylase (DLAT) foci and reduced adrenal ferredoxin 1 (FDX1 xpression.^[^ ^2^, ^16^ ^]^ Cu/TI treatment induced an average of 4.7 DLAT foci per cell, while other treatments did not induce DLAT foci in 4T1 cells (Figure 3c; and Figure S16, Supporting Information). Western blot results showed that Cu/TI treatment reduced FDX1 expression by 63.7% over PBS control but Cu‐NCP and TI‐NCP treatments had no impact on FDX1 expression (Figure 3e; and Figure S17, Supporting Information). These results show that Cu/TI efficiently induces cell death by targeting mitochondria, inducing oxidative stress, and causing cuproptosis.

### 2.4 Cu/TI Induces Release of DAMPs, Promotes DC Maturation, and Downregulates PD‐L1 Expression in Cancer Cells

**Figure 4:**
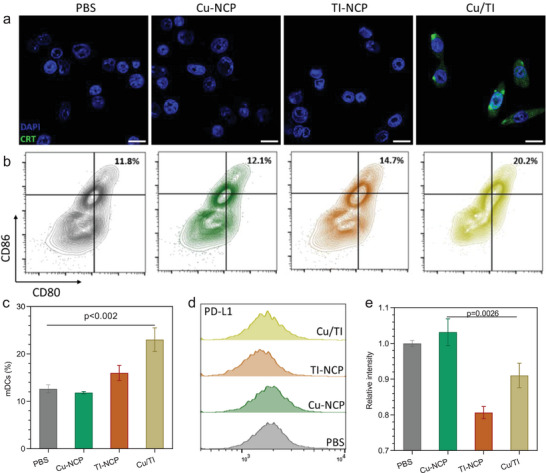
a) CRT expression (green signals) of 4T1 cells after different treatments. Scale bars are 25 µm. b) Flow cytometry analysis and c) quantification of mature DCs after coculturing with 4T1 cells that have received different treatments. d) Representative flow cytometry data and e) relative intensity of PD‐L1 expression of 4T1 cells after different treatments. Data are represented as mean ± SD, *n* = 3 in (c–e). One‐way ANOVA with Tukey's correction for statistical significance.

Cancer cells undergoing mitochondrial oxidative damage can initiate immunogenic cell death (ICD) to present DAMPs.^[^ ^17^ ^]^ Cu/TI treatment showed higher calreticulin (CRT) expression in 4T1 cells (**Figure** **4**a) and release of high mobility group box 1 (HMGB1) (Figure S18, Supporting Information) and adenosine triphosphate (Figure S19, Supporting Information) from 4T1 cells. Cu/TI treatment also greatly elevated the secretion of heat shock proteins (HSP70) in 4T1 cells (Figure S20, Supporting Information). The release of DAMPs from Cu/TI‐treated 4T1 cells induced dendritic cell (DC) maturation, increasing the proportion of mature DCs (CD80^+^CD86^+^) to 23.0% from 12.6% for the PBS group. The proportions of mature DCs were 11.8%, and 16.0% for Cu‐NCP and TI NCP groups, respectively (Figure 4b,c).

While Cu‐NCP treatment slightly elevated the PD‐L1 level by 1.03‐fold over the PBS control, TI‐NCP and Cu/TI treatments efficiently downregulated PD‐L1 expression to 0.81‐ and 0.91‐folds of the PBS group, respectively (Figure 4d,e). These results were consistent with the western blot results (Figure 3f; and Figure S17, Supporting Information). Taken together, Cu/TI treatment not only induces ICD to stimulate DC maturation but also downregulates PD‐L1 expression, thereby offering potential to activate the adaptive immune response in vivo.

### 2.5 Cu/TI Prolongs Blood Circulation to Accumulate in Tumors In Vivo

**Figure 5:**
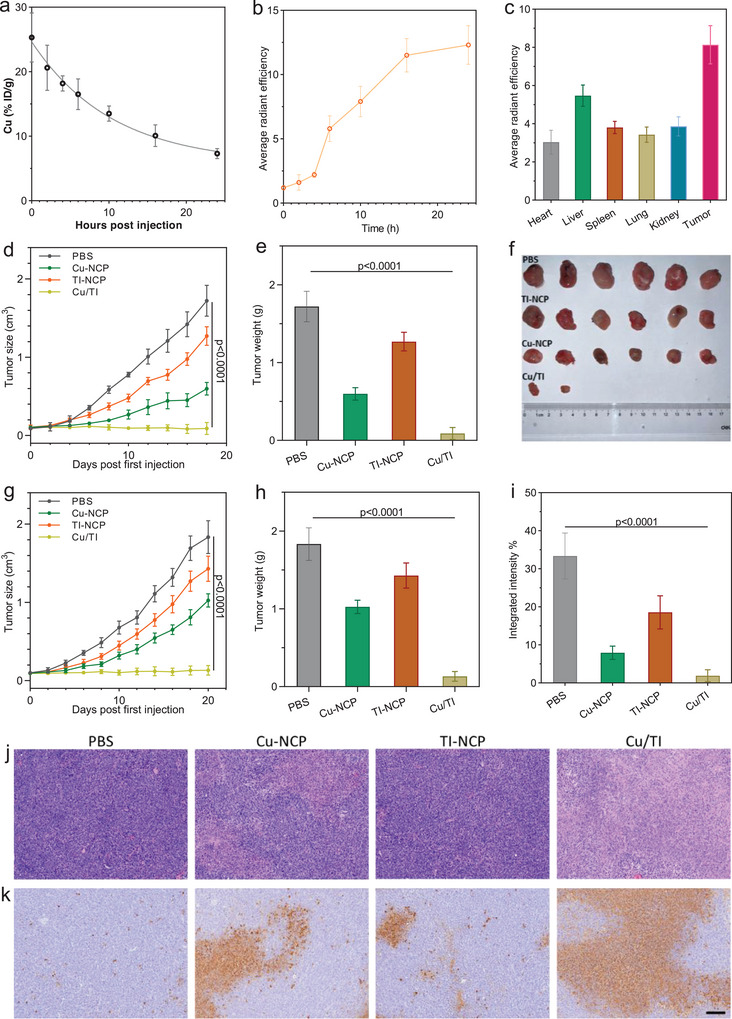
a) Time‐dependent Cu concentrations in the plasmas after i.v. injection of Cu/TPP‐pyro. b) Time‐dependent average pyro radiant efficiency in the tumors. c) Average pyro radiant efficiency in different organs 24 h post i.v. injection of Cu/TPP‐pyro NCP in 4T1 tumor‐bearing mice. Units are ×10^8^ [p s^−1^ cm^−2^/sr^−1^]/[µW cm^−2^]. d) Growth curves and e) Weights of CT26 tumors after different treatments. f) Photo of CT26 tumors at the endpoint after different treatments. g) Growth curves and h) Weights of 4T1 tumors after different treatments. i) Integrated metastasis tumor intensity in lungs in 4T1 tumor bearing mice after different treatments. j) Representative H&E staining and k) TUNEL staining of 4T1 tumors after different treatments. Scale bar is 100 µm. Data are represented as mean ± SD, *n* = 6 in (a–e) and (g–i). One‐way ANOVA with Tukey's correction for statistical significance.

We tested cytotoxicity of Cu/TI in normal cells. Cu/TI inhibited HEK293T cell growth with an IC_50_ of 2.98 ± 0.29 µm (Figure S21 and Table S5, Supporting Information). We used Cu/TPP‐pyro, a control NCP particle with Cu encapsulated in the core and TPP‐pyro on the shell as a surrogate to assess the pharmacokinetics (PK) and biodistribution of Cu/TI in 4T1 tumor‐bearing mice. The half‐life of Cu was determined to be 7.23 ± 1.63 h by ICP‐MS analyses of plasma samples collected at different timepoints (**Figure** **5**a; and Table S6, Supporting Information). Meanwhile, the half‐life of TPP‐pyro was determined to be 9.57 ± 2.21 h by ex vivo imaging of plasma samples collected at different timepoints (Figure S22 and Table S7, Supporting Information). In vivo imaging of 4T1 tumor‐bearing mice following the injection of Cu/TPP‐pyro showed gradually increased TPP‐pyro signals in the tumors over a 24 h period (Figure 5b; and Figure S23, Supporting Information), with higher ex vivo signals in the tumors compared to other major organs at 24 h postinjection (Figure 5c; and Figure S24, Supporting Information). The tumor‐targeting ability of NCPs in tumors can potentially enhance antitumor efficacy while minimizing systemic toxicity of the treatments.^[^ ^18^ ^]^

### 2.6 Cu/TI Elicits Robust Antitumor Effects

We tested the antitumor efficacy of Cu/TI in a CT26 colorectal carcinoma mouse model and a 4T1 triple negative breast cancer mouse model. Seven days post subcutaneous implantation of CT26 or 4T1 cells, tumor‐bearing mice with average tumor volume of 100 mm^3^ were randomly grouped (*n* = 6) and intravenously injected with PBS, Cu‐NCP, TI‐NCP, or Cu/TI at an equivalent dose of 1 mg kg^−1^ Cu or/and 6 mg kg^−1^ IOX1 once every 3 days for 3 doses.

In CT26 tumor‐bearing mice, Cu/TI treatment regressed tumors with a tumor growth inhibition (TGI) index of 94.8% and 4 out of 6 mice being tumor free. Cu‐NCP and TI‐NCP treatments failed to control the tumors with TGIs of 65.3% and 26.2% respectively (Figure 5d–f). The bodyweights of all the experimental mice remained within the healthy range, suggesting no obvious systematic toxicity of these treatments (Figure S25, Supporting Information).

In 4T1 tumor‐bearing mice, Cu/TI treatment efficiently inhibited the growth of the tumors with a TGI of 92.8% and 3 out of 6 mice being tumor free. Cu‐NCP and TI‐NCP treatments failed to control the tumors with TGIs of 44.1% and 22.1%, respectively (Figure 5g,h). The 4T1 tumors were also sectioned for hematoxylin and eosin (H&E) as well as TUNEL staining. Cu/TI‐treated tumors displayed greatly reduced cell nuclei signals (Figure 5j), and greatly increased DNA fragments (Figure 5k). Pulmonary metastasis rate in 4T1 tumor‐bearing mice was greatly reduced from 33.4% in the PBS group to 1.9% in the Cu/TI group (Figure 5i; and Figure S26, Supporting Information). Mouse bodyweights did not show obvious decrease in all treated mice (Figure S27, Supporting Information), and the examination of major organs showed no damage, except for a small amount of tumor metastasis in the hearts, livers, and kidneys in the PBS group (Figure S28, Supporting Information). The detailed histopathological analysis also showed that the major organs displayed normal histopathological characteristics after Cu/TI treatments (Figure S29, Supporting Information). Alanine aminotransferase, aspartate aminotransferase, and creatinine levels in the sera of 4T1 tumor‐bearing BALB/c mice extracted 24 h post the last injection were within the normal ranges (Figure S30, Supporting Information). We found that the macrophage cell line RAW 264.7 was less sensitive to the Cu/TI treatment compared to normal cells (Figure S31 and Table S8, Supporting Information), indicating nontoxicity of Cu/TI to immune cells. The hemolysis test results also demonstrated the hemocompatibility of Cu/TI at the therapeutic dose (Figure S32, Supporting Information).

### 2.7 Cu/TI Elicits an Antitumor Immune Response

**Figure 6:**
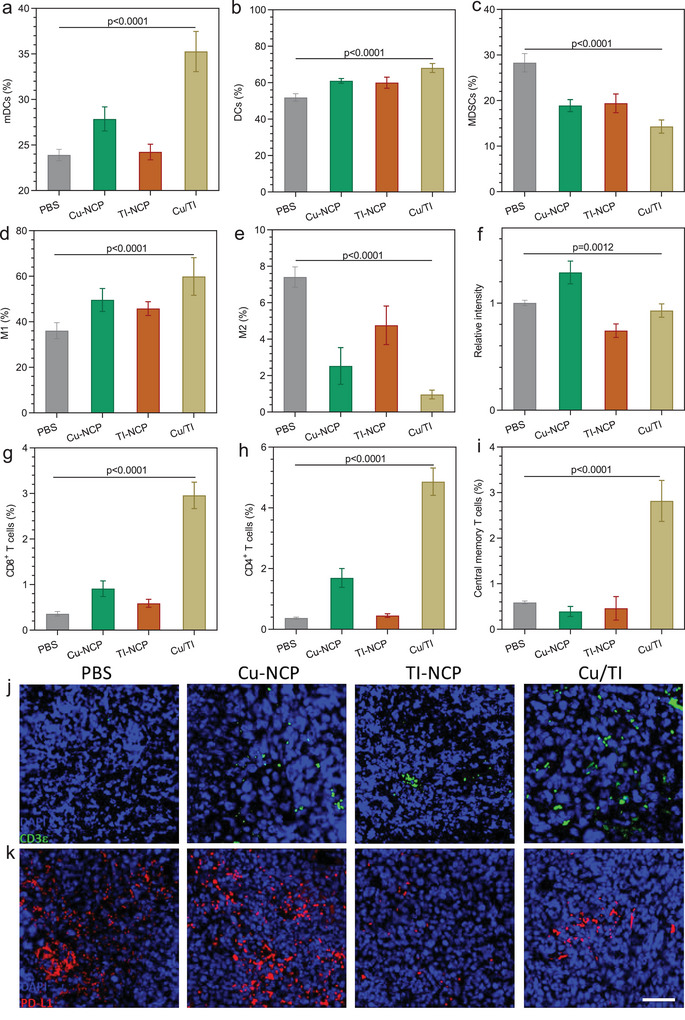
a) Quantification of mature DCs in TDLNs of 4T1 tumor‐bearing mice with different treatments. Quantification of b) DCs, c) MDSCs, d) M1, and e) M2 cells in the tumor tissue f) Relative PD‐L1 expression of tumor cells, Quantification of g) CD8^+^ T cells and h) CD4^+^ T cells in the tumors of 4T1 tumor‐bearing mice with different treatments. i) Percentage of central memory T cells in spleens of 4T1 tumor‐bearing mice with different treatments. j) CD3ε and k) PD‐L1 expression in the treated tumors by immunofluorescence staining. Scale bar is 50 µm. Data are represented as mean ± SD, *n* = 6 in (a–i). One‐way ANOVA with Tukey's correction for statistical significance.

We profiled the immune cells in the tumor draining lymph nodes (TDLNs) and tumors of 4T1 tumor‐bearing mice after different treatments. In the TDLNs, the population of mature DCs increased to 35.3% in the Cu/TI group compared to 23.9%, 27.9%, or 24.2% in PBS, Cu‐NCP, or TI‐NCP group, respectively (**Figure** **6**a; and Figures S33–S34, Supporting Information). In the tumors, the population of DCs increased to 68.1% in the Cu/TI group compared to 51.9%, 61.0%, or 60.0% in PBS, Cu‐NCP, or TI‐NCP group, respectively (Figure 6b; and Figures S35–S36, Supporting Information). Additionally, Cu/TI treatment reduced myeloid‐derived suppressor cells (MDSCs) in the tumors to 14.3% from 28.3% in the PBS group (Figure 6c; and Figure S37, Supporting Information). Antitumor M1 macrophages in the tumors increased from 36.1% for the PBS group to 49.6% for the Cu/TI group while pro‐tumor M2 macrophages decreased from 7.4% for the PBS group to 0.96% for the Cu/TI group (Figure 6d,e; and Figure S38, Supporting Information). The M1/M2 ratio greatly increased from 4.9 for the PBS group to 62.4 for the Cu/TI group (Figure S39, Supporting Information).

Cu‐NCP treatment increased PD‐L1 expression in the tumors by 28.6% over the PBS control, while Cu/TI treatment reduced PD‐L1 expression in the tumors to 0.93‐fold of the PBS group (Figure 6f; and Figures S40–S41, Supporting Information). Immunofluorescence (IF) staining of the tumors after different treatments showed consistent PD‐L1 expression results to the flow cytometric analysis results (Figure 6k). The percentage of CD8^+^ T cells reached 2.96% in the Cu/TI group, compared to 0.36%, 0.91%, or 0.59% for PBS, Cu‐NCP, or TI‐NCP group, respectively (Figure 6g; and Figure S42, Supporting Information). The percentage of CD4^+^ T cells also increased to 4.86% for the Cu/TI group, compared to 0.37%,1.69%, or 0.45% for PBS, Cu‐NCP, or TI‐NCP group, respectively (Figure 6h; and Figure S43, Supporting Information). IF staining of Cu/TI treated tumors also showed more CD3ε signals than other groups (Figure 6j). Notably, Cu/TI treatment also induced anti‐tumor memory effect. In the spleens, the percentage of central memory T cells increased to 2.82% for the Cu/TI group, compared to 0.59%, 0.39%, or 0.46% for PBS, Cu‐NCP, or TI‐NCP group, respectively (Figure 6i; and Figures S44–S45, Supporting Information).

**Figure 7:**
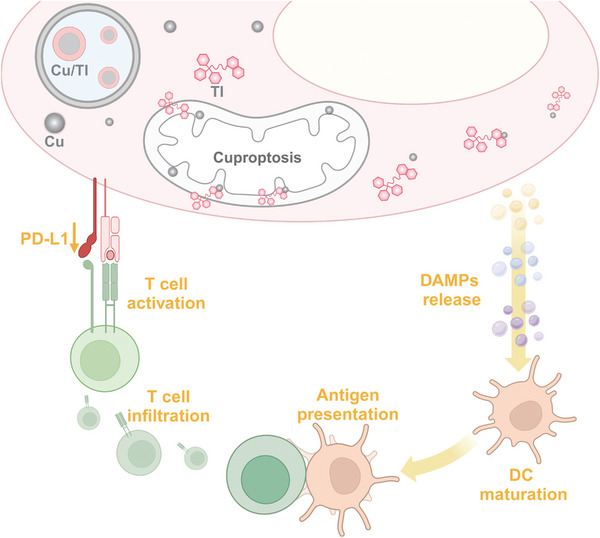
Scheme depicting the proposed antitumor mechanism of Cu/TI. In tumors, TI transports Cu to mitochondria to induce cuprotosis of and downregulate PD‐L1 expression in cancer cells. Cu/TI promotes DC maturation by inducing ICD and facilitates antigen presentation with the released DAMPs, which further enhances antigen processing and presentation to naïve T cells. With PD‐L1 downregulation by TI, the primed T cells attack tumor cells for antitumor immunity. The figure was generated with BioRender.

Based on these findings, we propose the antitumor mechanism for Cu/TI in **Figure** **7**. After accumulation in tumors in vivo, TI facilitates the transport of Cu to the mitochondria of cancer cells to trigger cell cuproptosis and downregulate PD‐L1 expression. Cu/TI also enhances DC maturation by inducing ICD and the released DAMPs facilitate antigen presentation. Antigen processing and presentation to naïve T cells facilitate T cell priming, proliferation, and infiltration to the tumors, while the downregulation of the PD‐L1 immune checkpoint allows the primed T cells to attack tumor cells for antitumor immunity. With the increasing focus on exploring novel programmed cell death pathways like cuproptosis for cancer treatment,^[^ ^19^ ^]^ our study has uncovered a new method to precisely target mitochondria with molecular therapeutics that induce cuproptosis while simultaneously downregulating PD‐L1 expression.

## 3 Conclusion

In this work, we reported a mitochondria‐targeted core–shell NCP particle for simultaneous cuproptosis induction and PD‐L1 downregulation. With long blood circulation and high tumor accumulation, Cu/TI not only reversed the poor immunogenicity of established tumors by inducing ICD, but also decreased PD‐L1 expression in cancer cells. As a result, Cu/TI efficiently promoted the infiltration and activation of cytotoxic T lymphocytes to inhibit the growth of colorectal carcinoma and triple‐negative breast cancer in mouse models without causing side effects. Considering the chemical diversity, high loading capacity, and intrinsic biodegradability, NCPs provide a promising platform for organelle‐targeted multidrug delivery.

## Conflict of Interest

W.L. is the founder of Coordination Pharmaceuticals, Inc., which licenses the NCP technology from the University of Chicago. R.R.W. is a scientific advisor to Coordination Pharmaceuticals. All other authors declare no competing financial interest.

## Author Contributions

Y.L. and W.L. conceived the project. Y.L. performed the experiments and analyzed the results. Y.L. J.L., R.R.W., and W.L. wrote the manuscript.

## Supporting information

Supporting Information

## Data Availability

The data that support the findings of this study are available from the corresponding author upon reasonable request.
