# Supplementary material for: Mitochondria‐Targeted Multifunctional Nanoparticles Combine Cuproptosis and Programmed Cell Death‐1 Downregulation for Cancer Immunotherapy
Source: Adv Sci (Weinh). 2024 Jul 16;11(35):2403520. doi: 10.1002/advs.202403520 (PMC11425249; doi:10.1002/advs.202403520)
Supplement: Supplementary file 1 — Supporting Information [file ADVS-11-2403520-s001.pdf]

Supporting information

Mitochondria-Targeted Multifunctional Nanoparticles Combine Cuproptosis and Programmed Cell Death-1 Downregulation for Cancer Immunotherapy

*Youyou Li, Jing Liu, Ralph R. Weichselbaum, and Wenbin Lin**

Email: wenbinlin@uchicago.edu

Materials and Methods

All starting materials were purchased from Sigma-Aldrich and Fisher (USA), unless otherwise noted, and used without further purification. Transmission electron microscopy (TEM) was carried out on a TECNAI Spirit instrument. Particle sizes were measured via dynamic light scattering (DLS) on a Malvern Nano Series Zeta-Sizer. Flow cytometry data were collected on an LSR-Fortessa 4-15 (BD Biosciences) and analyzed by FlowJo software. Confocal laser scanning microscope (CLSM) images were collected on Leica SP8 and analyzed with ImageJ software. ICP-MS results were obtained on a 7700 series ICP-MS (Agilent Technologies).

Cell Lines and Animals

Murine colon adenocarcinoma CT26 cells and murine metastatic triple-negative breast carcinoma 4T1 cells were purchased from the American Type Culture Collection (Rockville, MD, USA) and cultured in RPMI-1640 (Corning, USA) supplemented with 10% fetal bovine serum, 100 U/mL penicillin G sodium and 100 µg/mL streptomycin sulfate in a humidified atmosphere containing 5% CO_2 at 37 °C. BALB/c female mice (6 weeks, 18-22 g) were provided by Harlan Laboratories, Inc (USA). The study protocol (ACUP 72408) was reviewed and approved by the Institutional Animal Care and Use Committee (IACUC) at the University of Chicago [D16-00322 (A3523-01)]. The Human Tissue Resource Center at the University of Chicago provided histology related services for this study.

Synthesis and characterization of NCPs. Cu-bare particles were synthesized as the previously reported methods.[1-2] Briefly, an aqueous mixture of Na_3PO_4 (100 µL, 5 mg/mL) and 1,2-dioleoyl-sn-glycero-3-phosphate (DOPA) (150 µL, 200 mg/mL) in a 5 mL surfactant system (0.3 M Triton X-100 and 1.5 M 1-hexanol in cyclohexane) was sonicated for 1 min. Another aqueous solution of $CuCl_2$ (200 µL, 300 mg/mL) in 5 mL of the same surfactant system was stirred vigorously. The $CuCl_2$ solution was added dropwise to the Na_3PO_4 solution and stirred at room temperature for 10 min. Thirty milliliters of ethanol were added. DOPA-capped Cu bare particles were obtained by centrifugation at 12,000 ×g at 25 °C for 15 min. The resulting particles were washed with ethanol three times and redispersed in tetrahydrofuran

for lipid coating. The copper concentration was quantified by ICP-MS. For the preparation of NCPs, a THF solution of Cu-bare particle, TI (200 uL, 16 mM) or TPP-pyro (200 uL, 16 mM), DSPE-PEG_{2000} (300 µL, 5 mg/mL), DOPC (420 µL, 5 mg/mL), and cholesterol (84 µL, 5 mg/mL) were added dropwise to 1 mL of 30% ethanol/water under vigorously stirring. The organic solvents (THF and ethanol) were completely evaporated under a nitrogen flow, and the resultant particles were diluted in 1.0 mL phosphate-buffered saline (PBS) for further use. The quantities of Cu and TI in Cu/TI particles were determined by ICP-MS and LC-MS, respectively. Cu-NCP particles were similarly prepared without the addition of TI or TPP-pyro.

Cytotoxicity. 4T1 cells were seeded in a 96-well plate (2×10^3 cells/well) and incubated with different concentrations of IOX1, $CuCl_2$, $FeCl_2$, $FeCl_3$, $ZnCl_2$, TI or NCPs for 24 h. Cell viability was determined by MTS assay.

PD-L1 Expression by flow cytometry. 2×10^5 4T1 cells were seeded in 6-well plates and incubated with IOX1, $CuCl_2$, or NCPs for 24 h. The cells were collected and washed with PBS three times, then blocked by 5% FBS for 15 min and incubated with APC anti-mouse PD-L1 antibody (BioLegend) for 15 min and LIVE/DEAD™ Fixable Yellow Dead Cell Stain Kit (Thermo Fisher Scientific). The cells were washed three times and collected for flow cytometry analysis.

Subcellular localization. 4T1 cells (1×10^5 per well) were seeded in petri dishes and incubated with Cu/TPP-pyro NCP for different lengths of time. After washing with PBS three times, the cells were stained with 100 nM MitoTracker™ Red CMXRos (Thermo Fisher Scientific) for 15 min and then washed with PBS three times again before imaging under CLSM.

Mitochondrial Cu quantification. 4T1 cells (3×10^5 per well) were seeded in 6-well plates and after incubation for 24 hours, NCPs with equivalent doses of 1 µM Cu and/or 25 µM TI were added. After different time intervals, cells were collected and counted, then mitochondria were extracted by Mitochondria Isolation Kit (Thermo Fisher Scientific) and the Cu amount was quantified by ICP-MS.

Cell death. 4T1 cells (3×10^5 per well) were seeded in 6-well plates and incubated with NCPs at an equivalent dose of 1 µM Cu or/and 25 µM TI. The cells were harvested and washed with PBS three times, and stained with Annexin V and PI for 15 min before analysis by flow cytometry.

ROS generation *in vitro*. 4T1 cells (1×10^5 per well) were seeded in 6-well plates and incubated with NCPs at an equivalent dose of 1 µM Cu or/and 25 µM TI for 24 h. The cells were washed with PBS three times and stained with H_2DCFDA kit (Thermo Fisher Scientific). After washing with PBS three times, the stained cells were immediately imaged under CLSM.

Mitochondrial membrane potential change. 4T1 cells (1×10^5 per dish) were seeded in petri dishes and incubated with NCPs at an equivalent dose of 1 µM Cu or/and 25 µM TI for 24 h. After washing with PBS three times, the cells were stained with 10 µM JC-1 for 15 min. Then, the cells were washed with PBS three times to remove excess JC-1 and immediately observed by CLSM.

DLAT Foci analysis. 4T1cells (1×10^5 per dish) were seeded in petri dishes and incubated with NCPs at an equivalent dose of 1 µM Cu or/and 25 µM TI for 24 h. The cells were collected and washed with

PBS three times. The cells were fixed with 4% paraformaldehyde (PFA), then blocked and permeabilized by 5% FBS + 0.2% Triton-X in PBS. Afterwards, the cells were incubated with DLAT antibody (13426-1-AP, Thermo Fisher Scientific) in 1% BSA at 4 °C overnight. The cells were washed with PBS three times and incubated with secondary antibodies (AF647 anti-rabbit IgG, Cell signaling technology) in 1% BSA at room temperature for 1 h. The cells were washed with PBS three times and stained by MitoTracker™ Red CMXRos (Thermo Fisher Scientific) and Hoechst (Thermo Fisher Scientific). Foci counter[3] (automatic background was used and tolerance setting was 75[4]) plugin was used to mask foci segmentation and counted the numbers of foci. The results were normalized to foci count per cell in each well.

Western blot. 4T1 cells were seeded in 6-well plates (3×10^5 per well) and incubated with NCPs at an equivalent dose of 1 μM Cu or/and 25 μM TI for 24 h. The cells were collected, lysed with RIPA with protease inhibitor (Thermo Fisher Scientific). The protein was quantified by BCA kit (Thermo Fisher Scientific), and separated by 4–12% NuPAGE Bis-Tris Mini Gels (Thermo Fisher Scientific). After transfer, the membranes were blocked, then incubated with primary antibodies against FDX1 (EPR4629, Abcam), PD-L1 (ab205921, Abcam), or GAPDH (Abcam). After washing with TBST, the membrane was incubated with HRP-linked secondary antibody anti-rabbit IgG (Cell Signaling Technology). Then the membrane was incubated with ECL Western Blotting Substrate (Thermo Fisher Scientific) and finally exposed under FluorChem R system (ProteinSimple).

CRT expression. 2×10^5 4T1cells were seeded in 6-well plates and incubated with NCPs at an equivalent dose of 1 μM Cu or/and 25 μM TI for 24 h. The cells were fixed with 4% PFA, and then stained with anti-CRT antibody (AF488 calreticulin antibody; Novus bio) in 1% BSA at 4 °C overnight. The cells were washed three times before observation under CLSM.

HMGB1 release. 4T1 cells were seeded in 6-well plates (3×10^5 per well) and incubated with NCPs at an equivalent dose of 1 μM Cu or/and 25 μM TI for 24 h. The media were collected after centrifugation and tested by HMGB1 Detection kit (Chondrex).

BMDCs maturation. Bone marrow-derived dendritic cells (BMDCs) were obtained from the marrow cavities of 6-week-old female C57BL/6 mice. They were cultured in RPMI-1640 medium containing GM-CSF (20 ng mL^{-1}) and IL-4 (10 ng mL^{-1}). A transwell system was utilized to study BMDCs maturation. Briefly, 4T1 cells (1×10^5 cells per well) and BMDCs (5×10^5 cells per well) were seeded in the transwells and 12-well plates, respectively, overnight. Afterwards, 4T1 cells were incubated with NCPs at an equivalent amount of 1 μM Cu or/and 25 μM TI for 24 h and then co-cultured with BMDCs for another 24 h. Then BMDCs were collected and stained with PE-eFluro610 anti-CD11c, PE/Cy7 anti-CD80 and APC anti-CD86. The population of matured DCs (mDCs, $CD11c^+CD80^+CD86^+$) was detected by flow cytometry.

HSP70 expression. 2×10^5 4T1cells were seeded in 6-well plates and incubated with NCPs at an equivalent dose of 1 μM Cu or/and 25 μM TI for 24 h. The cells were harvested and stained with HSP70 (AF647 HSP70 antibody; Novus bio) before analysis by flow cytometry.

***In vivo* antitumor efficacy.** CT26 cells (2×10^6) or 4T1 cells (1×10^6) were subcutaneously injected into the right flank regions of 6-week-old BALB/c mice. Seven days later, the tumor-bearing mice with

average tumor volume of 100 mm^3 were randomly grouped ($n = 6$) and intravenously injected with PBS, Cu NCP, TI NCP, or Cu/TI NCP at an equivalent dose of 1 mg/kg Cu or/and 6 mg/kg IOX1 once every 3 days (Q3D) for 3 doses. 24 hours after the final dose, sera were harvested to measure aspartate aminotransferase (AST), alanine aminotransferase (ALT), and creatinine with AST Activity Assay Kit (Sigma-Aldrich, USA), ALT Activity Assay Kit (Sigma-Aldrich, USA), and Creatinine Assay Kit (Sigma-Aldrich, USA), respectively. Tumor volumes were monitored daily and calculated as (width × length × width)/2. TGI was calculated as (1-average of tumor weights of treated group/ average of tumor weights of control group) ×100%. Mouse body weights were also monitored. Mice were sacrificed when the tumors of the control group reached 2 cm^3. Excised CT26 tumors were photographed. 4T1 tumors were sectioned for immunofluorescence staining, H&E staining and TUNEL staining. Hearts, lungs, livers, kidneys, and spleens of 4T1 tumor bearing mice were harvested and sectioned for H&E staining for systemic toxicity evaluation. Metastatic tumors were found in the lungs when the mice were dissected. Lung H&E slides were analyzed by QuPath software for evaluating tumor metastasis after different treatments.

Immune cell profiling. 4T1 tumor-bearing BALB/c mice were treated as described in the antitumor efficacy experiments. On day 12 post the first i.v. injection, tumors, spleens and tumor-draining lymph nodes (TDLNs) were harvested for immune cell profiling by flow cytometry. Briefly, tumors, spleens and TDLNs were digested with a cocktail of 1 mg/mL collagenase I + 1 mg/mL collagenase IV + 1 mg/mL DNase I in complete medium at 37 °C for 30 min. The tissues were ground and filtered through sterile cell strainers to obtain single cell suspensions. The cells were obtained by centrifugation (300 g, 5 min) at 4 °C and stained with LIVE/DEAD™ fixable yellow dead cell stain kit (ThermoFisher Scientific), then blocked with mouse anti-CD16/32 antibody at 4 °C for 5 min. The lymph node cells were stained with the following fluorochrome conjugated anti-mouse antibodies at 4 °C for 30 min: PE-eFluro610 anti-CD11c, PE/Cy7 anti-CD80 and APC anti-CD86. Tumor cells were stained by BV421 anti-CD45, FITC anti-CD11b, PE-eFluro610 anti-CD11c, PerCP-Cy5.5 anti-F4/80, APC anti-CD86, Pe-Cy7 anti-CD206 and APC-eFluor780 anti-Gr-1 (for DCs, MDSCs and macrophages profiling) or BV421 anti-CD45, PE-eFluor610 anti-CD3ε, APC-eFluor780 anti-CD8 and FITC anti-CD4 (for T cells profiling). Spleen cells were stained by PE-eFluor610 anti-CD3ε, PE anti-CD44 and APC anti-CD62L. All antibodies were obtained from eBioscience except those specifically noted. Compensation was calculated and results were analyzed by FlowJo software.

ATP release. 4T1 cells were seeded in 6-well plates (3×10^5 per well) and incubated with NCPs at an equivalent dose of 1 μM Cu or/and 25 μM TI for 24 h. The media were collected after centrifugation and tested by ATP Assay Kit (Sigma-Aldrich).

Biodistribution. 1 mg/kg Cu/TPP-pyro was intravenously dosed to 8-week-old 4T1 tumor-bearing mice. The mice were sacrificed at different time points. Plasma, tumors, and other organs were collected and imaged by IVIS using the Cy5.5 channel or for Cu quantification by ICP-MS.

Hemolysis test. Fresh mouse blood was thoroughly washed with PBS to obtain erythrocytes. 5% erythrocytes in PBS (negative group), PBS solutions containing different concentrations of Cu/TI, and 0.1% Trion-X-100 water solution (positive group) were incubated at 37 °C for 1 hour. Then samples were centrifuged, and the absorbances for the supernatants were recorded at 540 nm (OD). Hemolysis rates were calculated as $(OD_{sample} - OD_{negative})/(OD_{positive}-OD_{negative}) \times100\%$.

Statistical analysis. All the statistical analysis was performed by One-way Repeated Measures ANOVA method with Tukey's honest significance test to determine whether the difference between each group was significant. The p values were defined as * $p < 0.05$, ** $p < 0.01$, *** $p < 0.001$, *** $p < 0.001$ in all the figures.

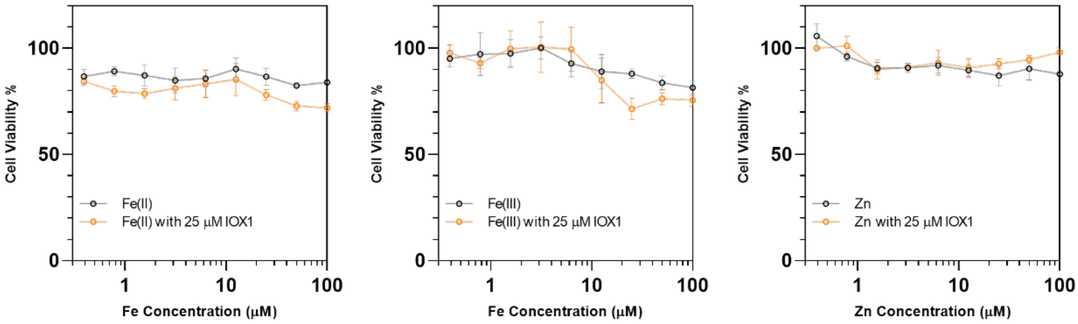

Figure S1. Cytotoxicity of Fe, Zn, and IOX1 under different conditions. Data are represented as mean ± SD, $n = 3$.

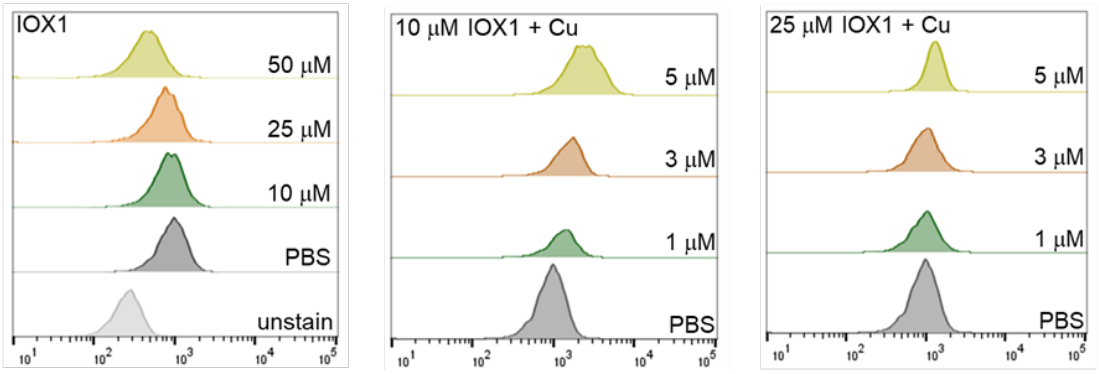

Figure S2. PD-L1 expression levels after different treatments by flow cytometry.

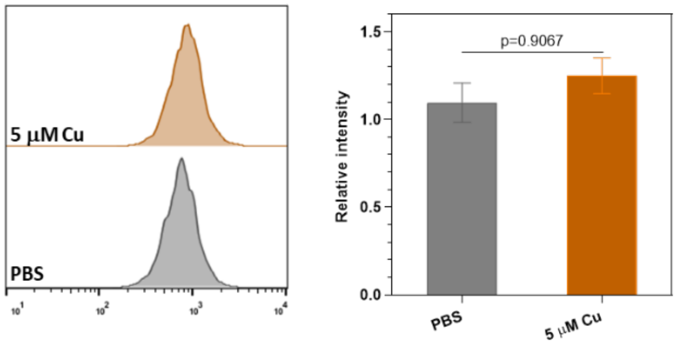

Figure S3. The PD-L1 expression level after $CuCl_2$ treatment by flow cytometry. Data are represented as mean ± SD, $n = 3$. One-way ANOVA with Tukey's correction for statistical significance.

Synthesis of IOX1. Acrolein diethyl acetal (2.9 mL, 1.5 eq) was added dropwise to 3-amino-4-hydroxybenzoic acid (2.0 g, 13.0 mmol) in 50 mL 4 M HCl solution. The mixture was stirred at room temperature for 30 min and then refluxed for 2 h. The resultant mixture was cooled to room temperature and adjusted to pH=9 by 28% aqueous ammonium hydroxide. The precipitate was filtered, and the

filtrate was acidified to pH=4 with 36% HCl solution to form precipitates. The dark sticky brown precipitates were then filtered. The solid was suspended in a mixed solution of water and acetone (1:1) before being filtered again to obtain the earthy yellow compound as the desired product. Yield: 1.1 g, 40.1%. ^{1}H NMR (400 MHz, DMSO-d_6): δ 12.74 (s, 1H), 9.48 (dd, J = 8.8, 1.6 Hz, 1H), 8.92 (dd, J = 4.1, 1.6 Hz, 1H), 8.26 (d, J = 8.3 Hz, 1H), 7.71 (dd, J = 8.8, 4.2 Hz, 1H), 7.14 (d, J = 8.3 Hz, 1H). HR-MS (ESI, positive ion mode): calcd for $[M+H]^+$, 190.0499; observed, 190.0505.

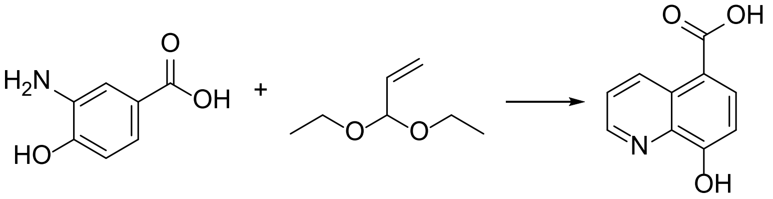

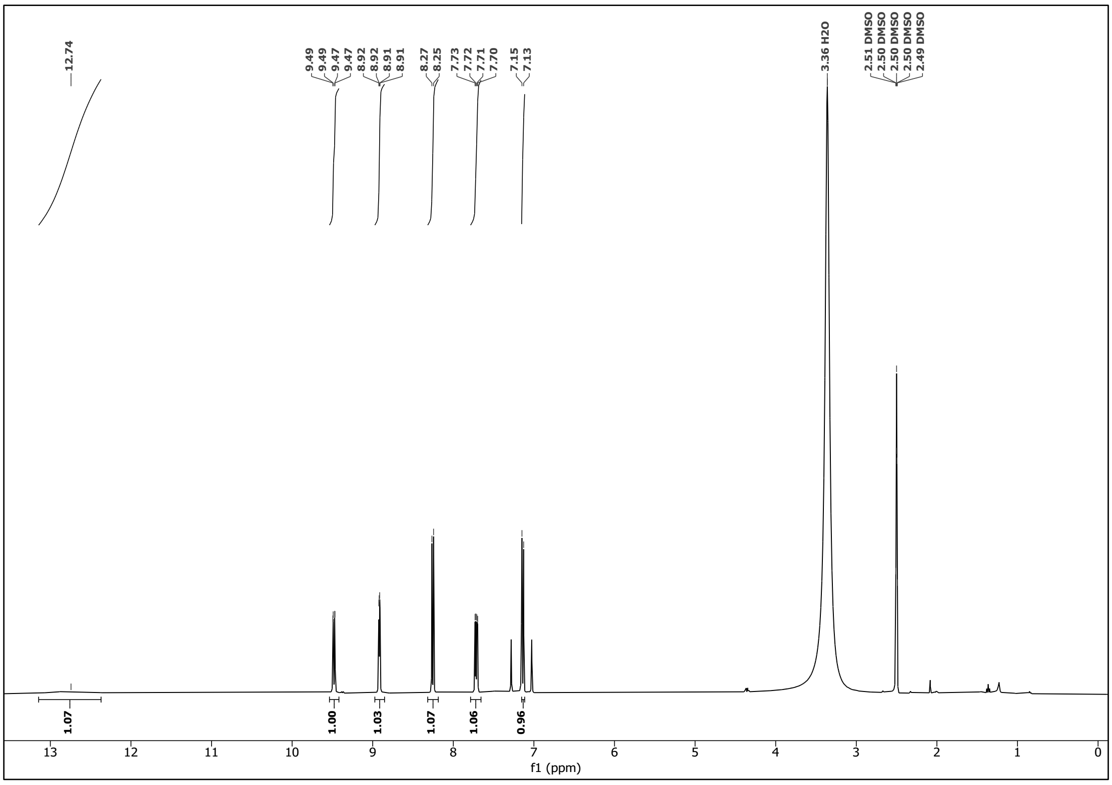

Figure S4. ^{1}H NMR spectrum of IOX1.

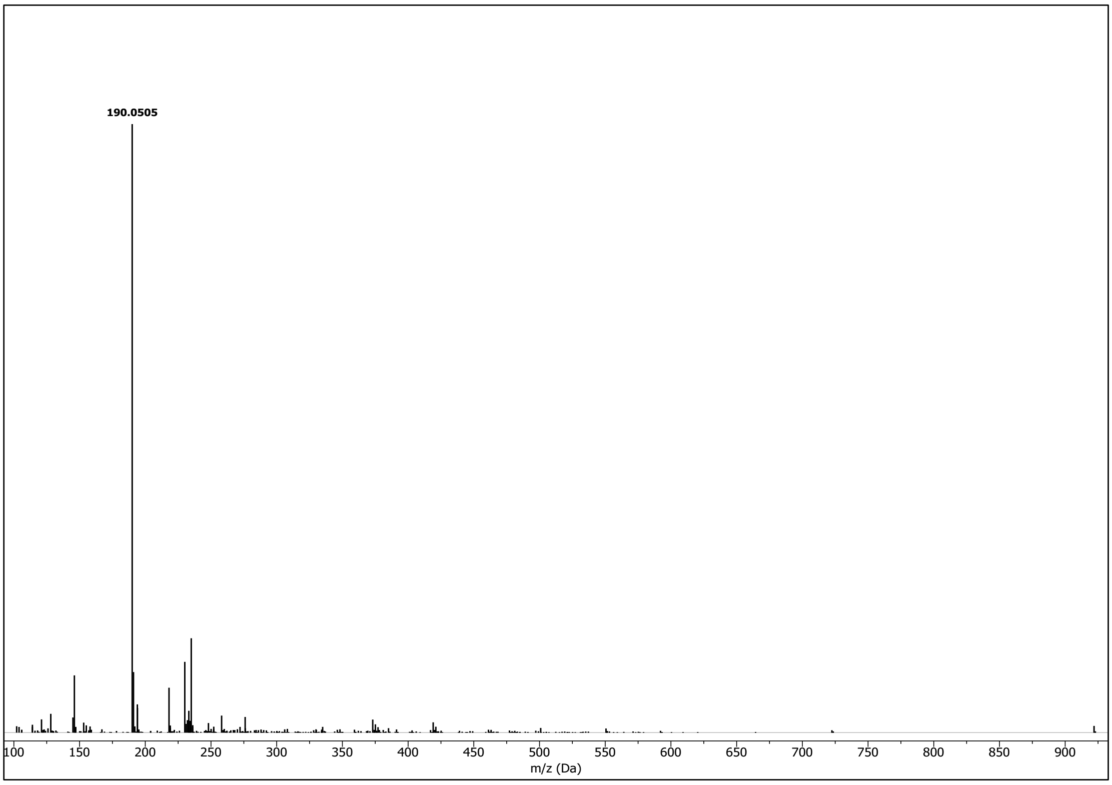

Figure S5. HR-MS of IOX1.

Synthesis of TPP-IOX1 (TI). IOX1 (100 mg, 0.53 mmol) was treated with EDCI (77.5 mg, 0.63 mmol, 1.2 eq) and DMAP (98.5 mg, 0.63 mmol, 1.2 eq) in 5 mL anhydrous dimethylformamide (DMF) at room temperature for 2 h. Then, (2-hydroxyethyl)triphenylphosphonium bromide (243.7 mg, 0.79 mmol, 1.5 eq) in 2 mL anhydrous DMF was added to the reaction under vigorous stirring. 24 h later, the mixture was precipitated using ethyl ether and purified by silica gel column chromatography to obtain the light-yellow product. Yield: 28 mg, 9.5%. ^{1}H NMR (400 MHz, DMSO-d_6): δ 9.48 (dd, *J* = 8.8, 1.7 Hz， 1H), 8.92 (dd, *J* = 4.1, 1.6 Hz,1H), 8.25 (d, *J* = 8.3 Hz, 1H), 7.89-7.70 (m,15H), 7.14 (d, *J* = 8.2 Hz, 1H), 3.80-3.74 (m, 4H). ^{13}C{^{1}H} NMR (101 MHz, DMSO-d_6): δ 167.53, 157.69, 148.03, 137.93, 134.77, 134.63, 134.60, 133.78, 133.68, 133.56, 130.02, 129.90, 128.08, 123.31, 119.61, 118.75, 116.38, 110.26, 54.52, 54.46, 25.14, 24.64. HR-MS (ESI, positive ion mode): calcd for $[M-Br]^+$, 478.157; observed, 478.167.

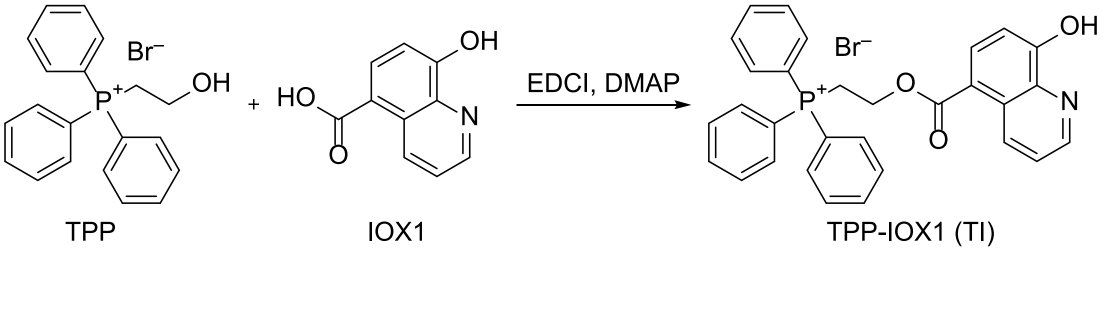

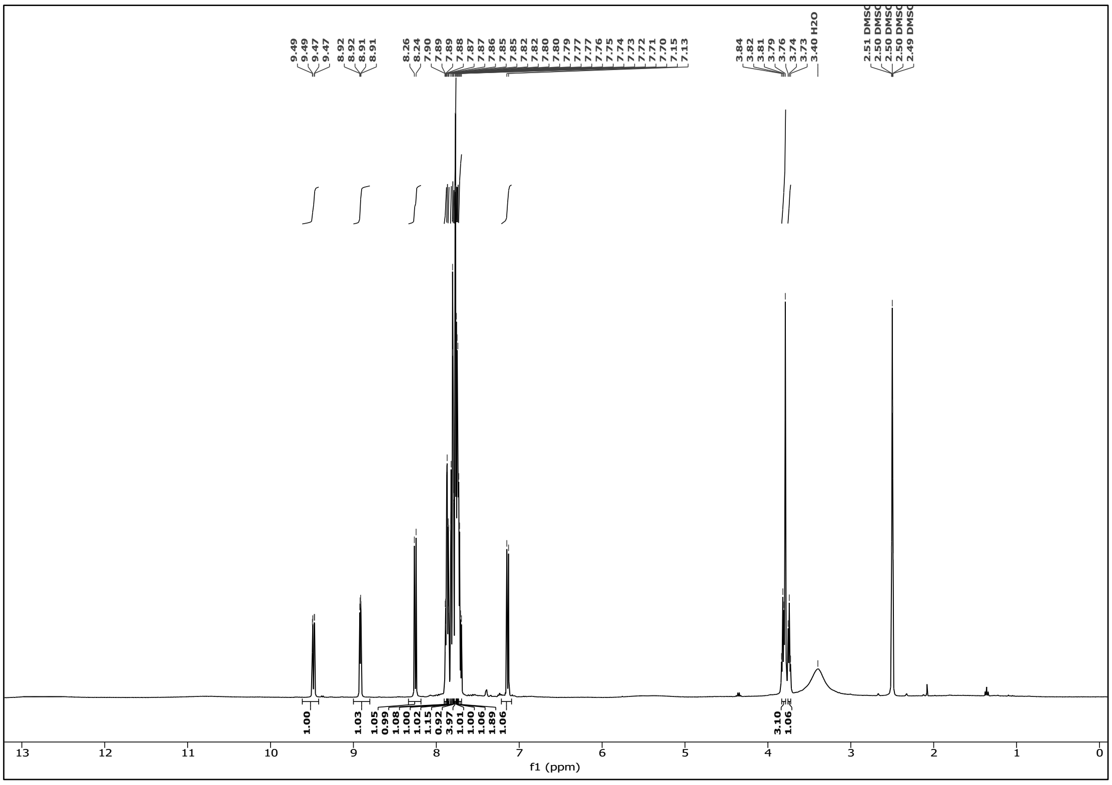

Figure S6. ^{1}H NMR spectrum of TI.

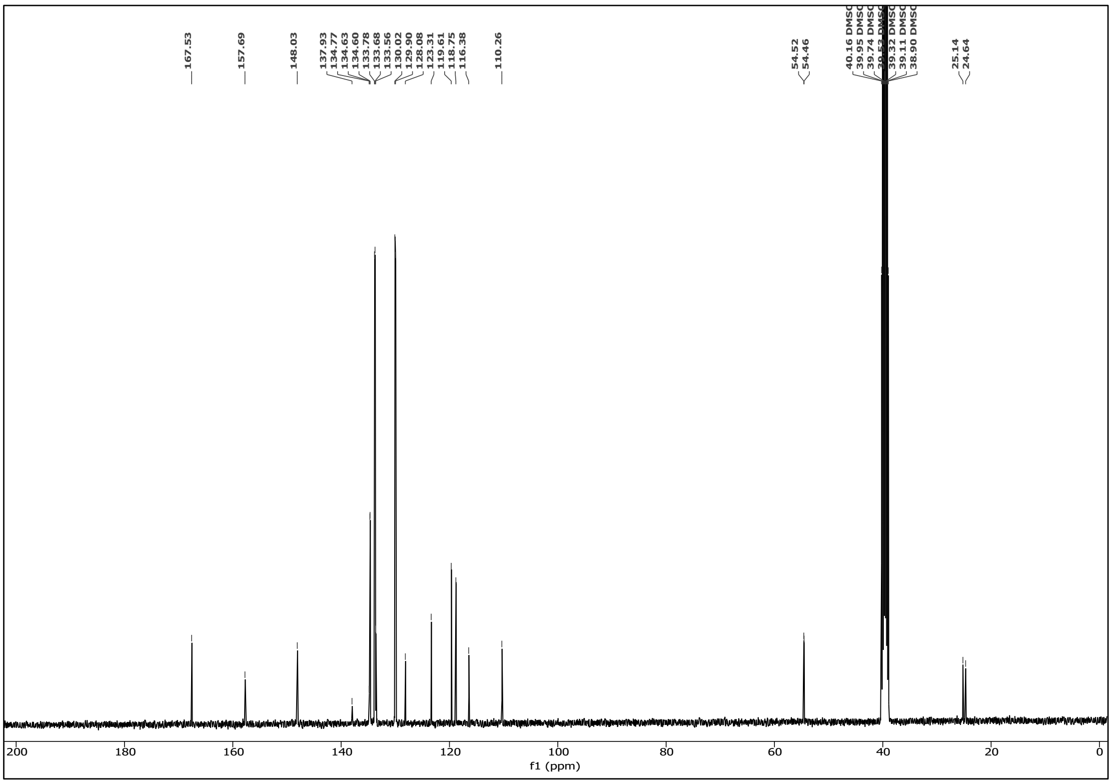

Figure S7. ^{13}C{^{1}H} NMR spectrum of TI.

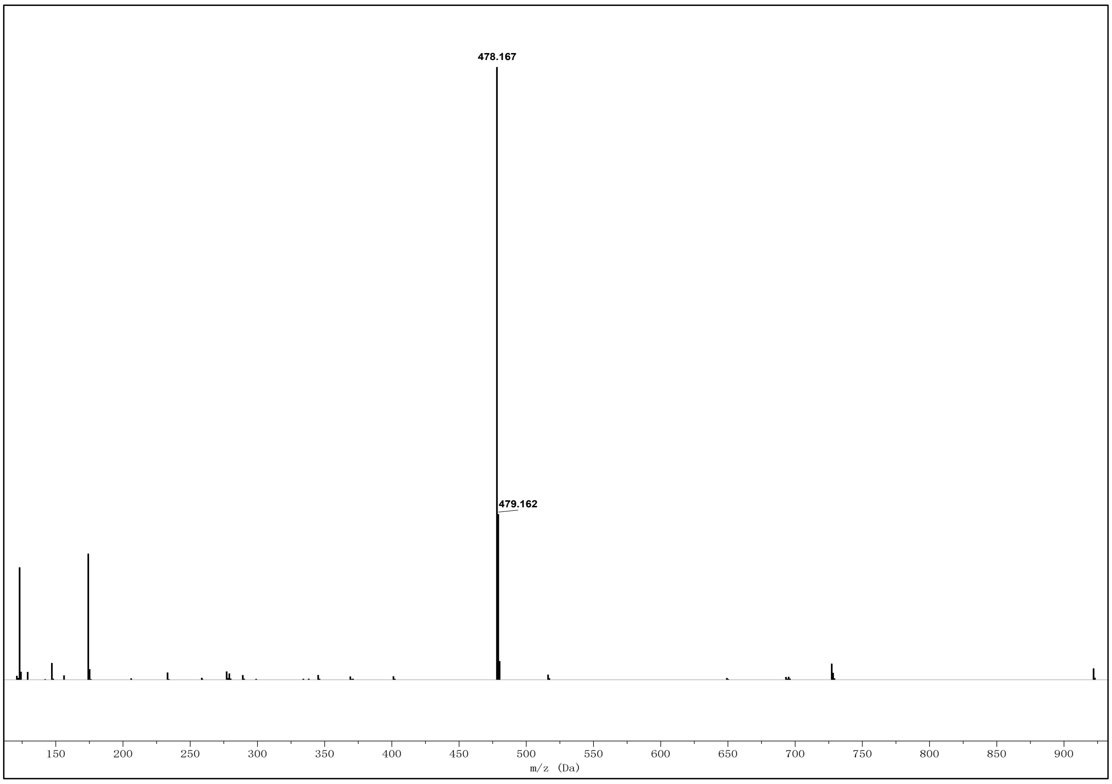

Figure S8. HR-MS of TI.

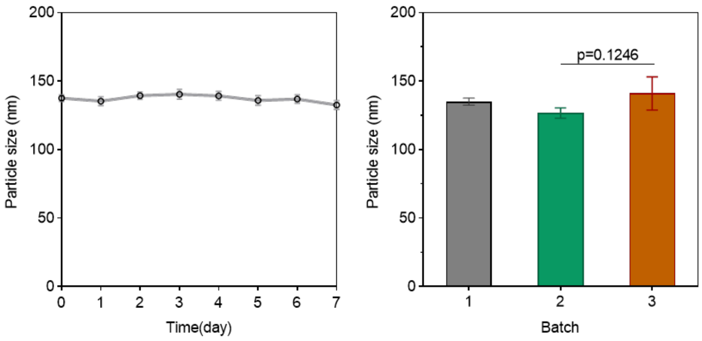

Figure S9. Particle sizes of Cu/TI during one-week incubation in PBS and particle sizes of three different batches of Cu/TI. Data are represented as mean ± SD, n = 3. One-way ANOVA with Tukey's correction for statistical significance.

Synthesis of TPP-pyro. Pyrolipid (100 mg, 0.19 mmol) was treated with EDCI (35.7 mg, 0.23 mmol, 1.2 eq) and DMAP (35.6 mg, 0.23 mmol, 1.2 eq) in 5 mL anhydrous DMF at room temperature for 2 h. Then, (2-hydroxyethyl)triphenylphosphonium bromide (87.9 mg, 0.29 mmol, 1.5 eq) in 2 mL anhydrous DMF was added to the reaction mixture under vigorous stirring. 24 h later, the mixture was precipitated using ethyl ether and purified by silica gel column chromatography to obtain the light-yellow product. Yield: 14.5 mg, 9.4%. ^{1}H NMR (400 MHz, DMSO-d_6) δ 9.76 (s, 1H), 9.68 (s, 1H), 9.09 (s, 1H), 8.27 (dd, J = 17.9, 11.7 Hz, 1H), 7.92 – 7.65 (m, 15H), 6.44 (d, J = 17.8 Hz, 1H), 6.17 (d, J = 11.6 Hz, 1H), 5.44 – 5.29 (m, 2H), 4.61 (q, J = 7.2 Hz, 1H), 4.44 (d, J = 10.3 Hz, 1H), 3.85 – 3.70 (m, 6H), 3.58 (s, 3H), 3.51(s,3H), 3.28 (s, 3H), 2.63–1.98 (m, 4H), 1.67 (m, 6H). ^{13}C{^{1}H} NMR (101 MHz, DMSO-d_6) δ

174.21, 173.56, 169.70, 168.06, 153.42, 148.29, 144.51, 138.18, 136.05, 135.15, 134.75, 134.57, 134.54, 134.11, 133.83, 133.71, 133.61, 130.30, 129.96, 129.84, 129.24, 128.90, 121.97, 119.55, 118.69, 103.37, 101.16, 98.27, 94.11, 54.48, 54.42, 52.56, 48.11, 40.15, 39.94, 39.73, 39.52, 39.31, 39.10, 38.89, 37.70, 30.82, 29.46, 26.85, 25.15, 24.65, 22.89, 18.86, 17.68, 11.99, 11.92, 10.93, 9.05. HR-MS (ESI, positive ion mode): calcd for $[M\text{-}Br]^{+}$, 823.377; observed, 823.386.

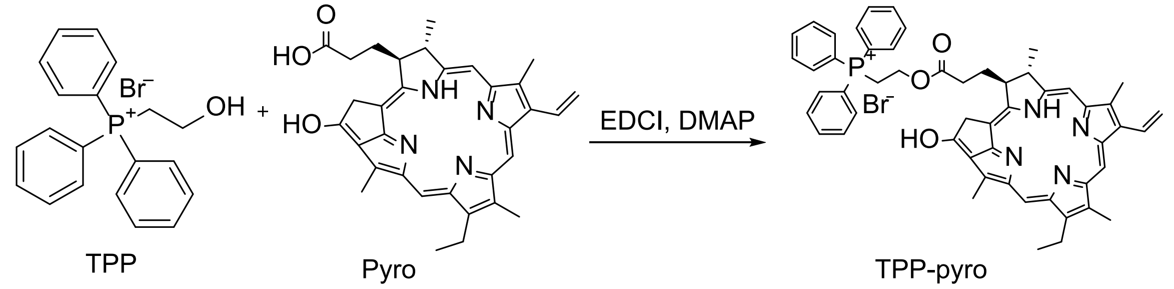

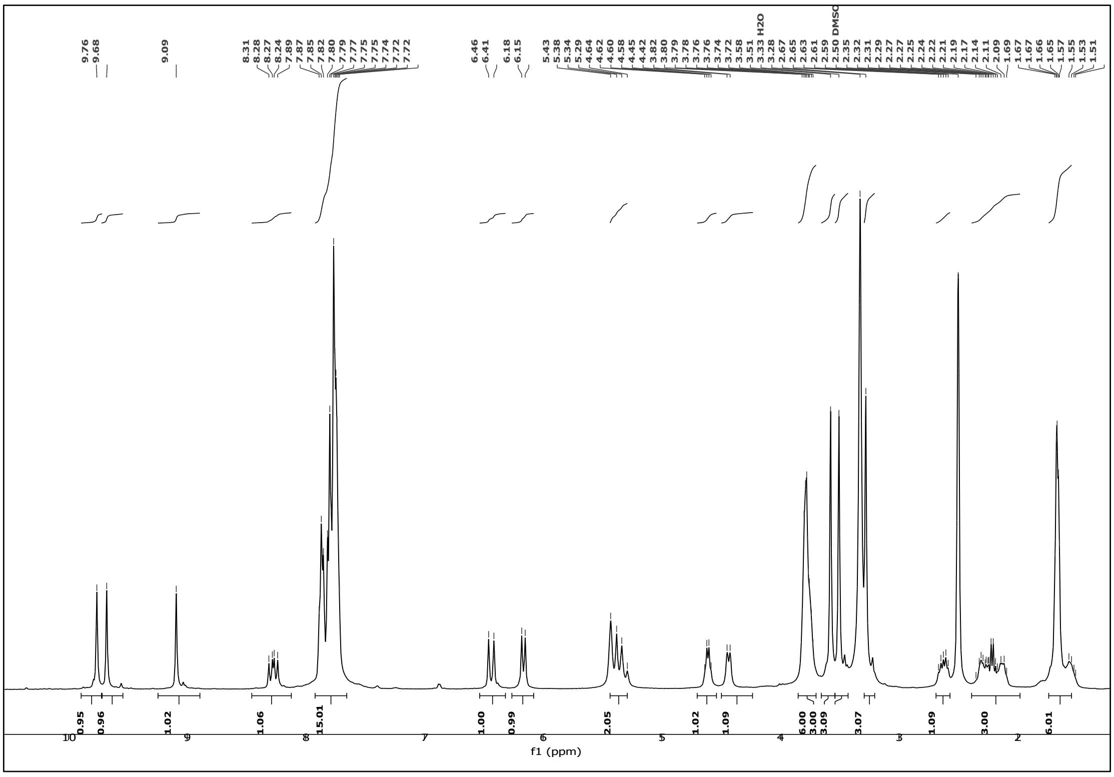

Figure S10. ^{1}H NMR spectrum of TPP-pyro.

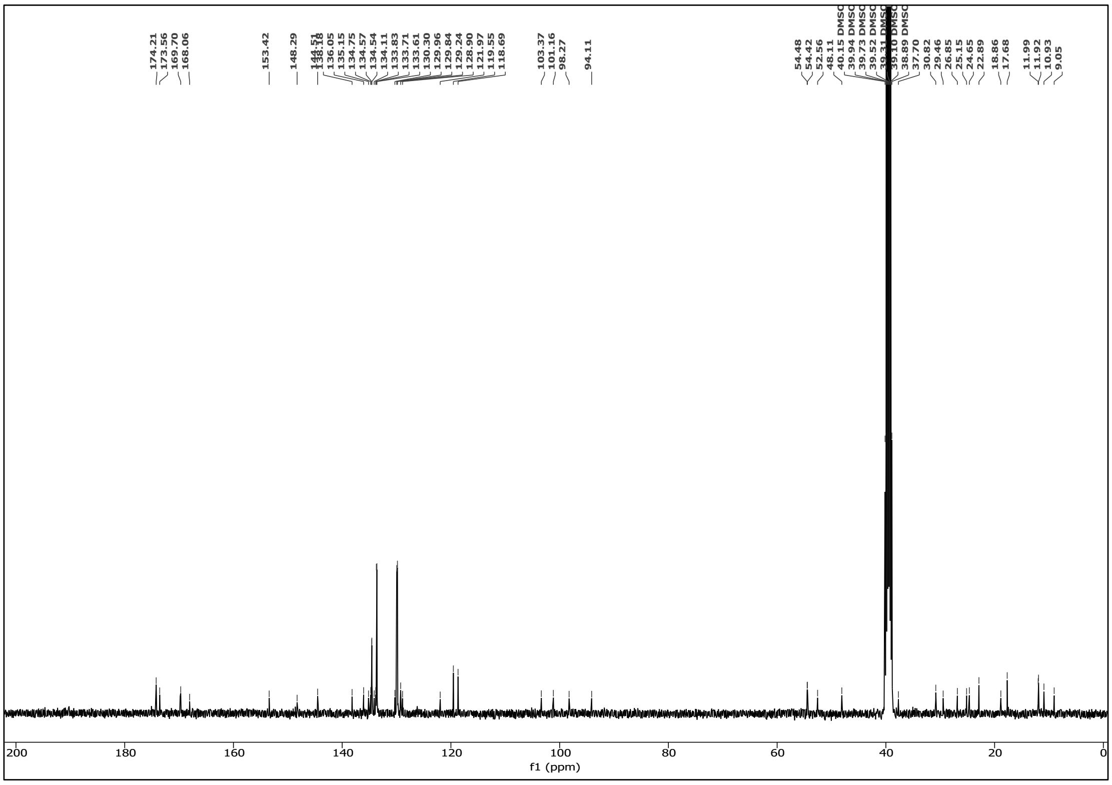

Figure S11. $^{13}C\{^{1}H\}$ NMR spectrum of TPP-pyro.

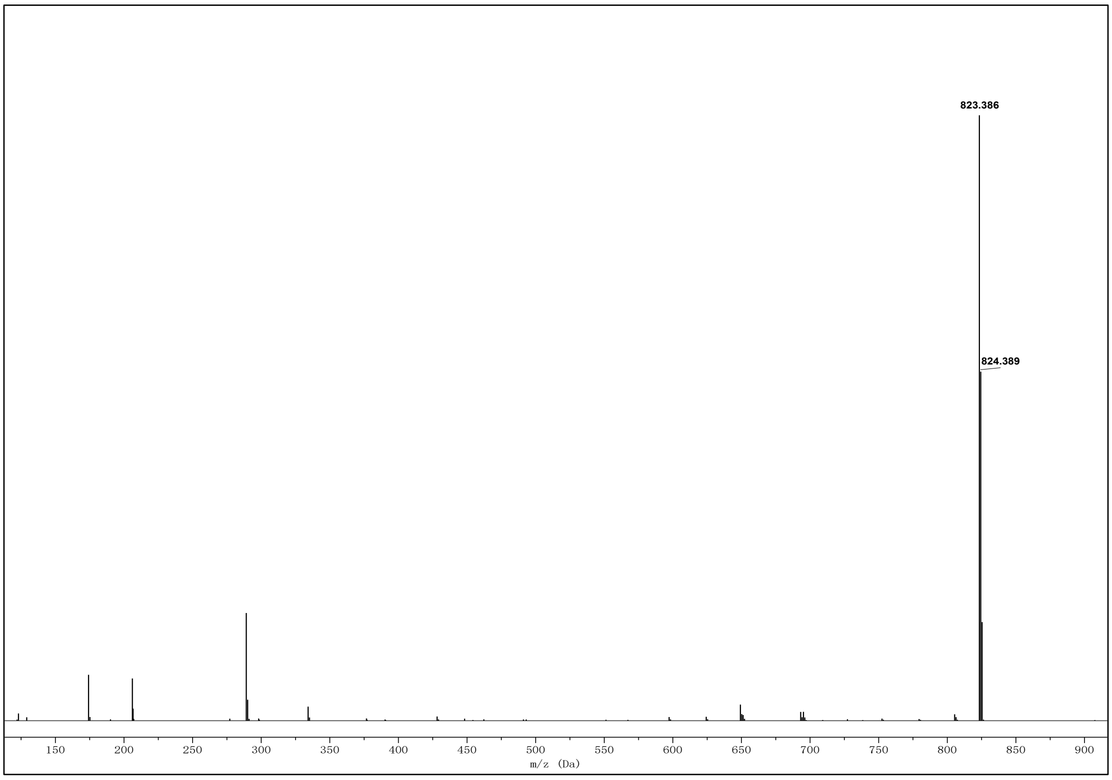

Figure S12. HR-MS of TPP-pyro.

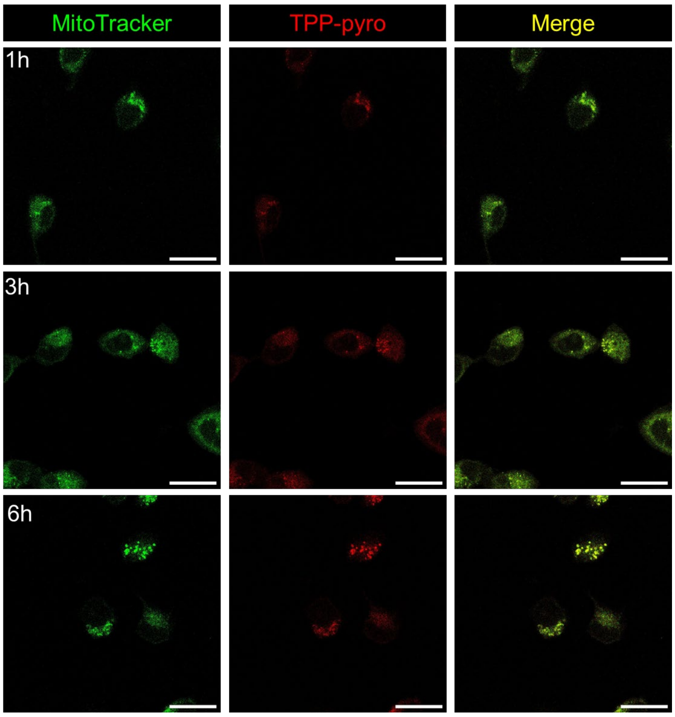

Figure S13. MitoTracker and TPP-pyro colocalization after incubating 4T1 cells with Cu/TPP-pyro for different lengths of time.

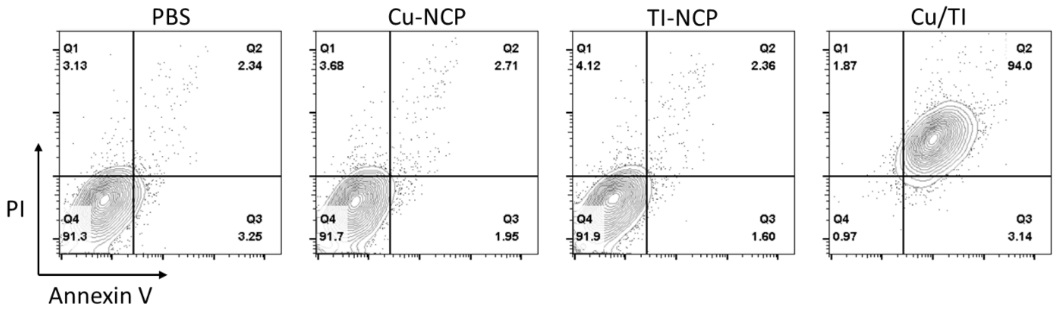

Figure S14. Representative flow cytometry data of 4T1 cell death after different treatments.

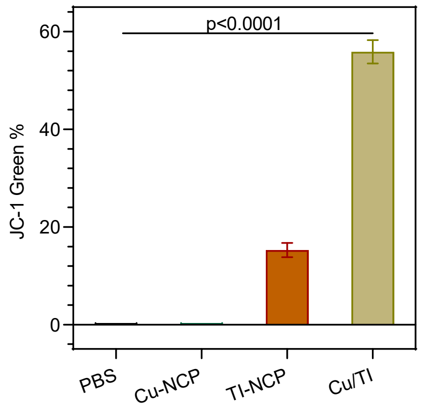

Figure S15. Mitochondrial membrane potential change represented by JC-1 green percentage in Figure 3c. Data are represented as mean ± SD, $n = 3$. One-way ANOVA with Tukey's correction for statistical significance.

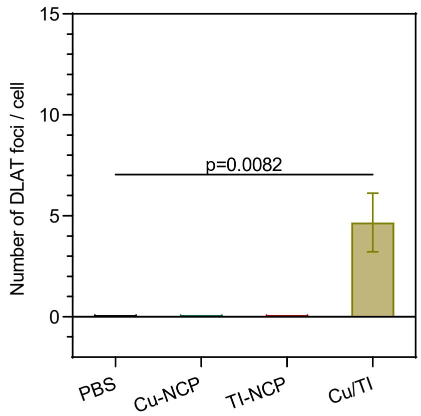

Figure S16. DLAT foci number per tumor cell by analyzing DLAT staining in confocal images in Figure 3d. Data are represented as mean ± SD, n = 3. One-way ANOVA with Tukey's correction for statistical significance.

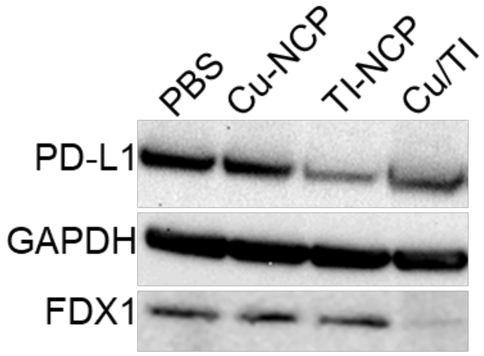

Figure S17. Representative western blot figures of PD-L1 and FDX1 protein expressions in 4T1 cells after different treatments.

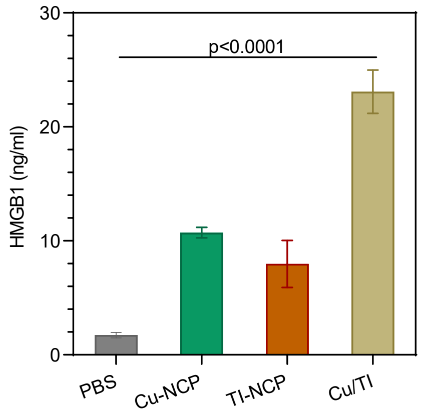

Figure S18. HMGB1 concentrations in 4T1 cell media after different treatments. Data are represented as mean ± SD, n = 3. One-way ANOVA with Tukey's correction for statistical significance.

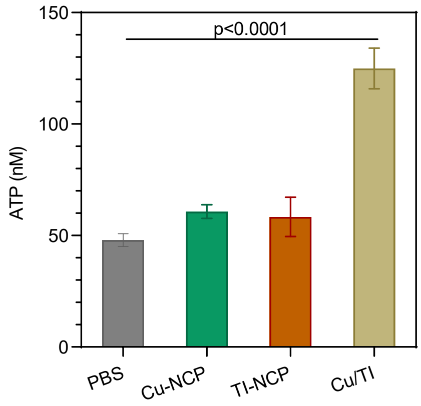

Figure S19. ATP secretion from 4T1 cells after different treatments. Data are represented as mean ± SD, $n = 3$. One-way ANOVA with Tukey's correction for statistical significance.

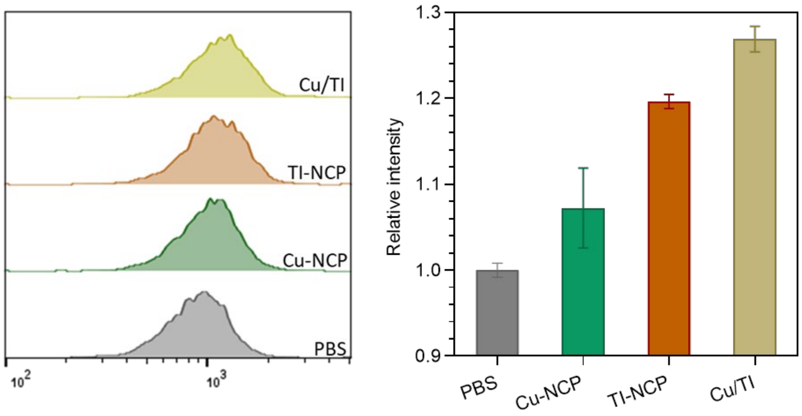

Figure S20. Representative flow cytometry data and relative intensities of HSP70 expressions in 4T1 cells after different treatments. Data are represented as mean ± SD, $n = 3$.

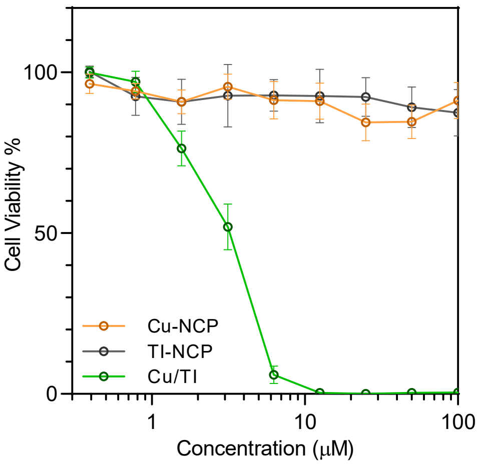

Figure S21. Cytotoxicity of different nanoparticles in HEK293T cells. For Cu/TI the IC_{50} is based on concentration of Cu (with 25 equivalents of TI). Data are represented as mean ± SD, $n = 3$.

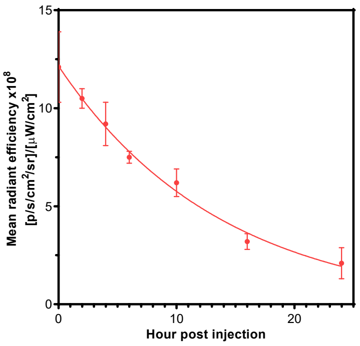

Figure S22. Time-dependent pyro fluorescence intensities in the plasmas after i.v. injection of Cu/TPP-pyro. Data are represented as mean ± SD, $n = 3$.

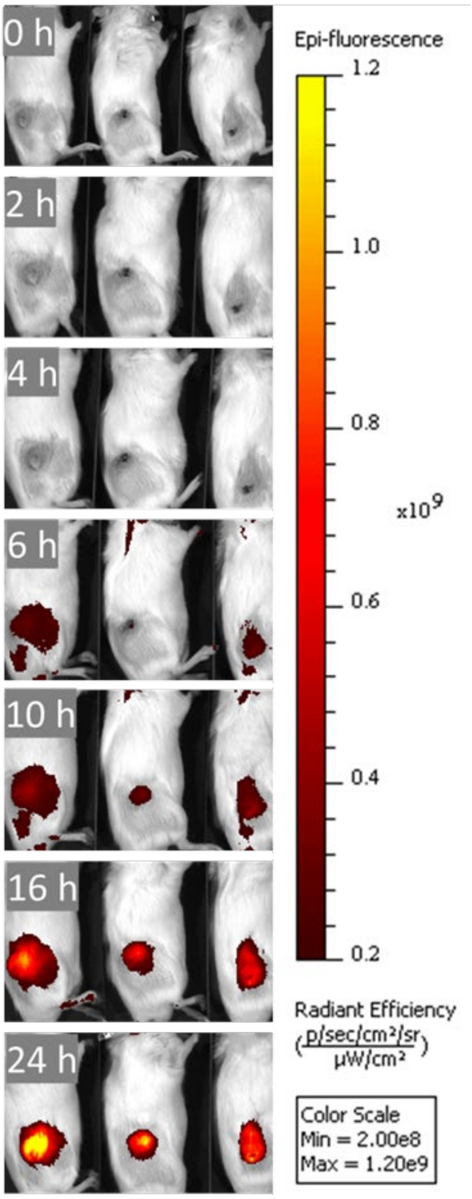

Figure S23. *In vivo* imaging of 4T1 tumor-bearing mice post i.v. injection of Cu/TPP-pyro.

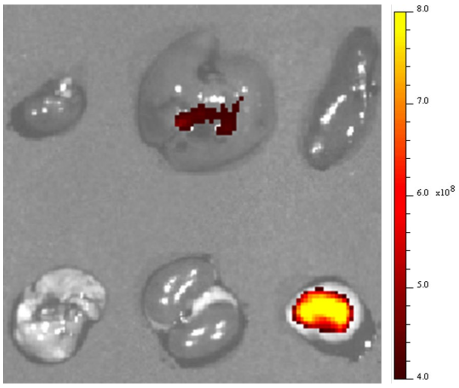

Figure S24. Representative image of pyro radiant efficiency in different organs 24 h post i.v. injection of Cu/TPP-pyro in 4T1 tumor-bearing mice. Units are $\times 10^8$ [p/s/cm^2/sr]/[μW/cm^2].

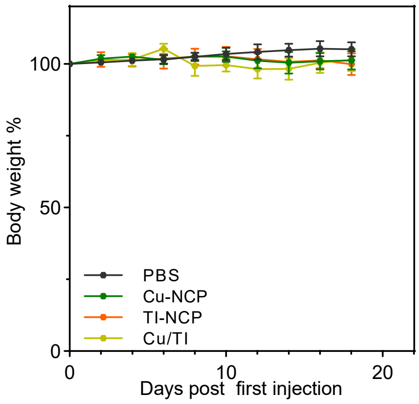

Figure S25. Relative bodyweights of CT26 tumor-bearing mice after different treatments. Data are represented as mean ± SD, $n = 6$.

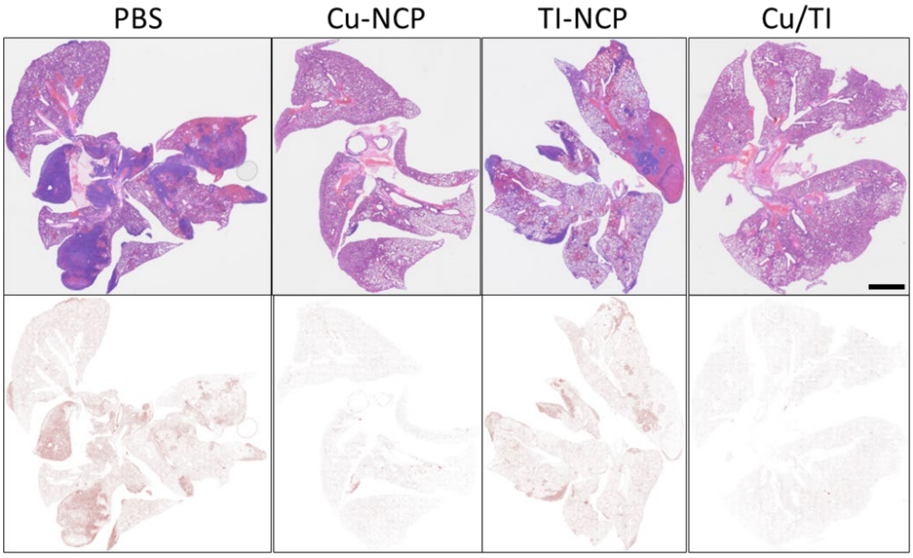

Figure S26. Representative H&E staining and lung metastasis in 4T1 tumor bearing mice after different treatments. Scale bar is 2 mm.

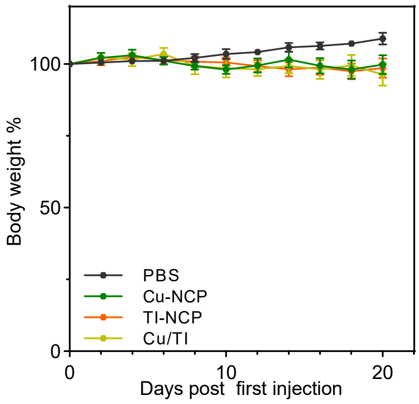

Figure S27. Relative bodyweights of 4T1 tumor-bearing mice after different treatments. Data are represented as mean ± SD, $n = 6$.

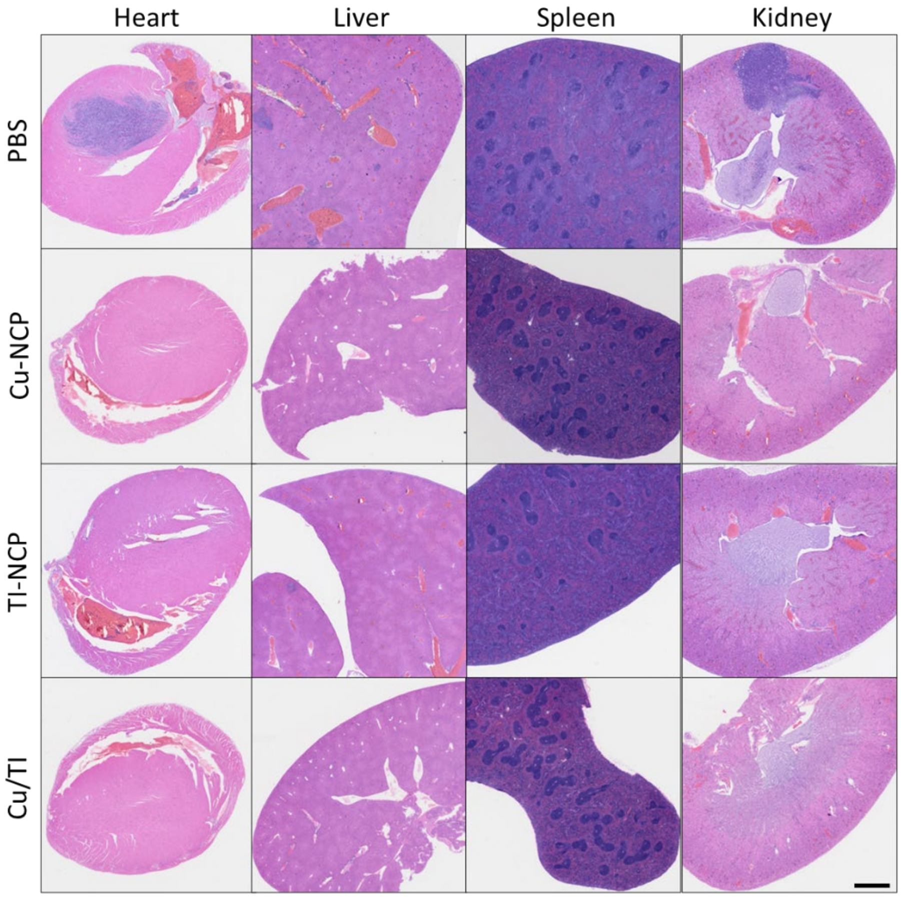

Figure S28. H&E staining of major organs in 4T1 tumor-bearing mice after different treatments. Scale bar is 1 mm.

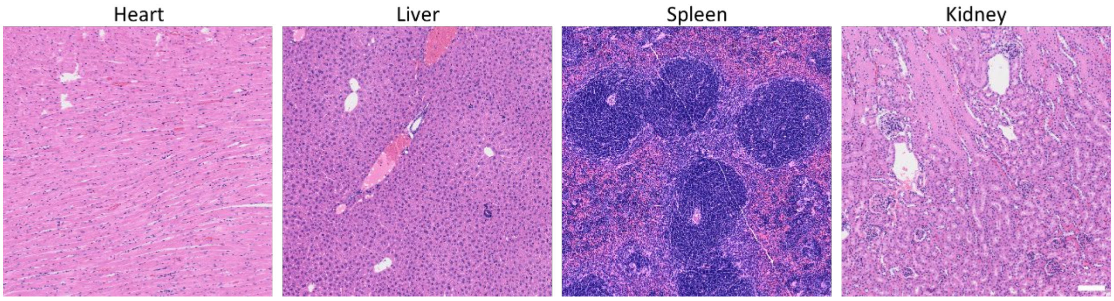

Figure S29. H&E staining of major organs in 4T1 tumor-bearing mice after Cu/TI treatments. Scale bar is 100 μm.

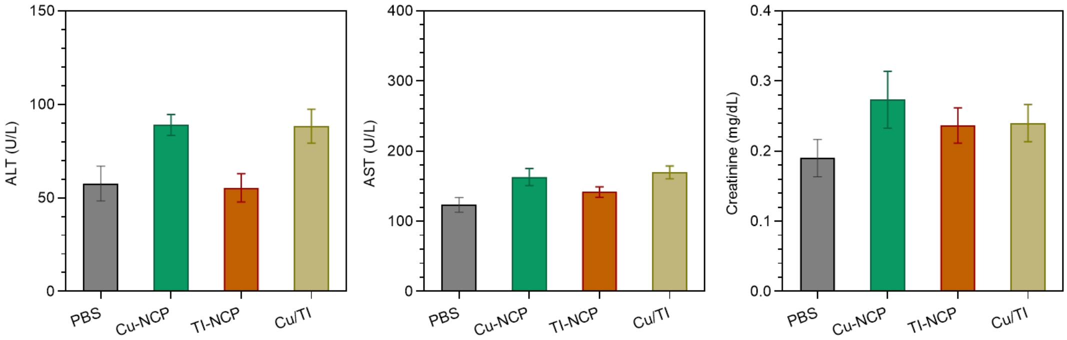

Figure S30. Serum ALT, AST, and creatinine levels from 4T1 tumor-bearing BALB/c mice 24 hours after the final dose. Normal ranges of BALB/c mice: 41-131 U/L for ALT, 55-352 U/L for AST, and 0.2-0.4 mg/dL for creatinine. Data are represented as mean ± SD, n = 3.

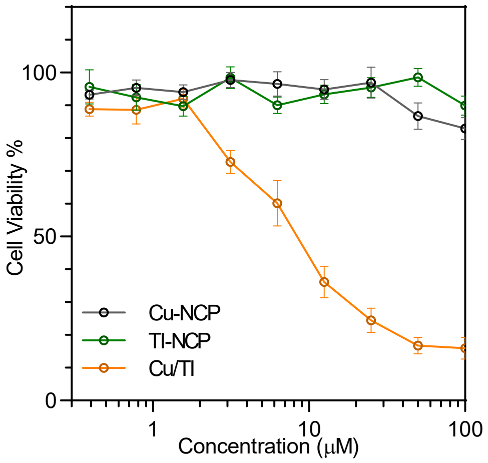

Figure S31. Cytotoxicity of different nanoparticles in RAW 264.7 cells. For Cu/TI the IC_{50} is based on concentration of Cu with 25 μM TI. Data are represented as mean ± SD, n = 3.

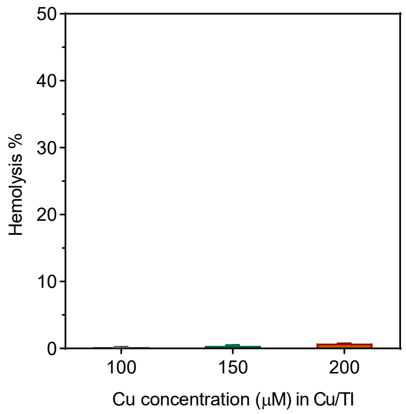

Figure S32. Hemolysis test of Cu/TI. Data are represented as mean ± SD, $n = 3$.

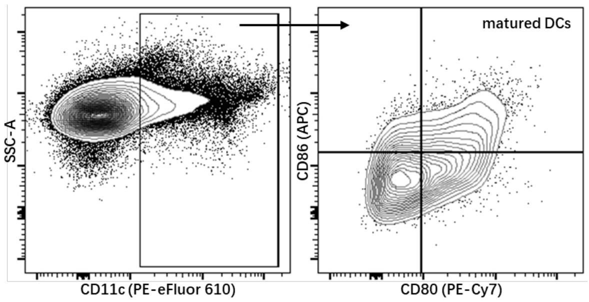

Figure S33. Gating strategies in single and live cells for matured DCs ($CD11c^+CD80^+CD86^+$) in the TDLNs.

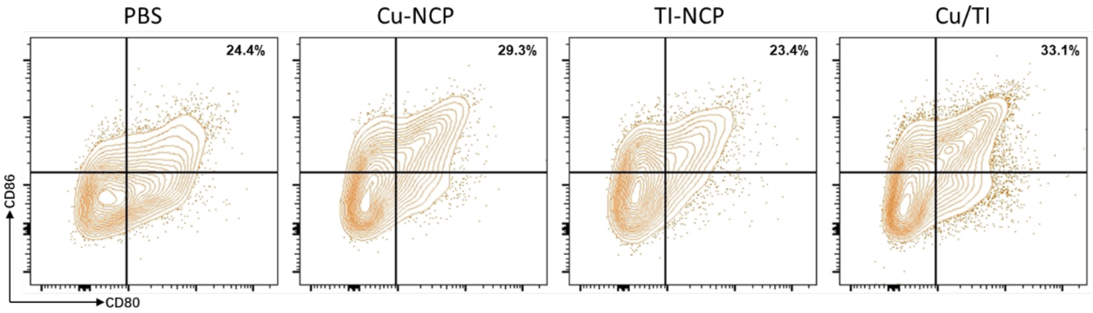

Figure S34. Representative flow cytometric analysis of matured DCs (mDCs, $CD11c^+CD80^+CD86^+$) in the TDLNs.

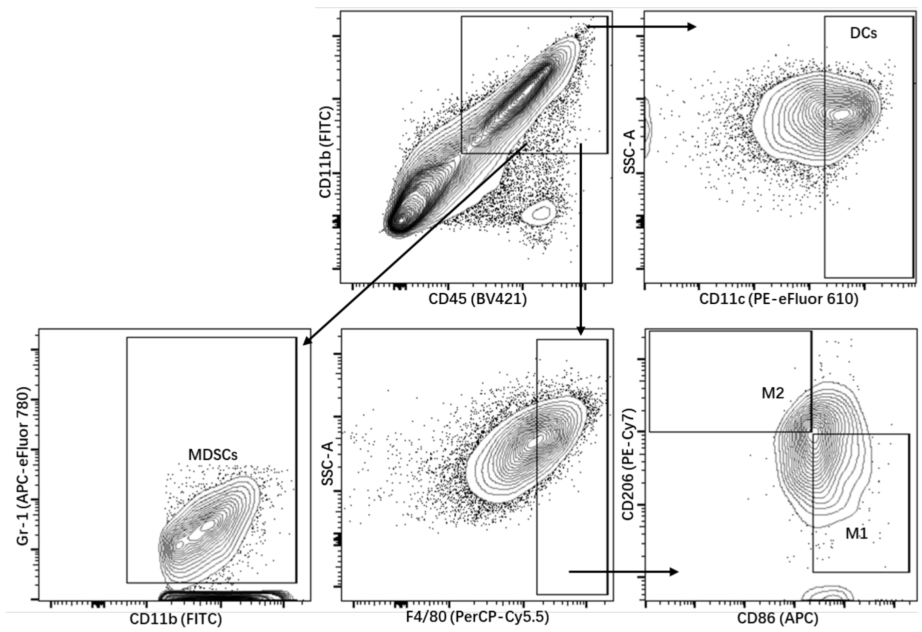

Figure S35. Gating strategies in single and live cells for DCs, MDSCs, M1 and M2 macrophages in the tumors.

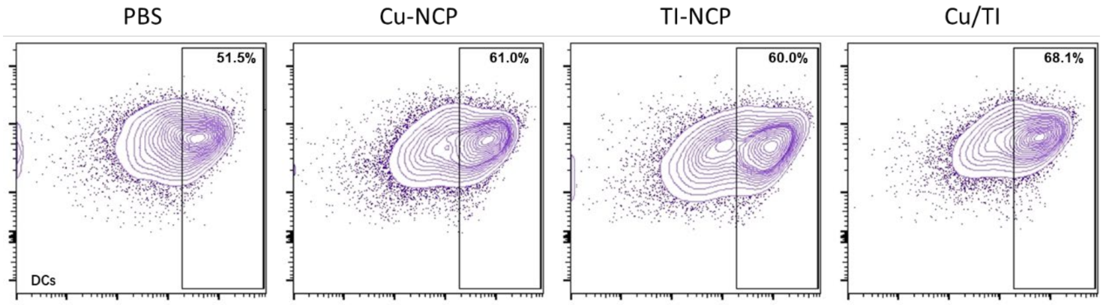

Figure S36. Representative flow cytometric analysis of DCs ($CD45^+CD11b^+CD11c^+$) in the tumors.

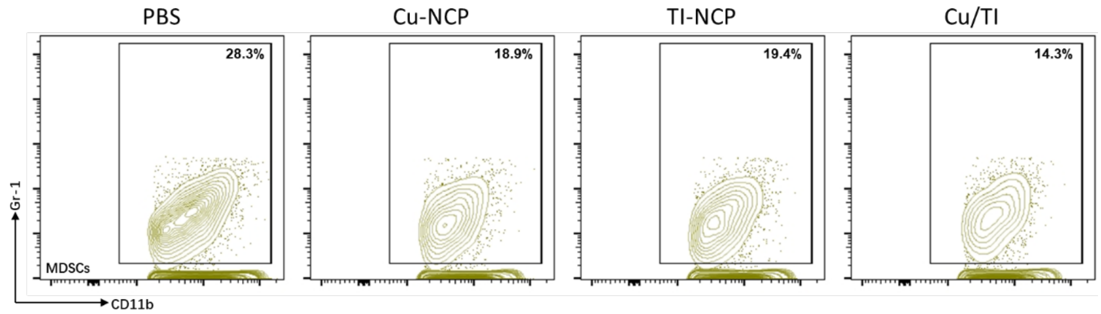

Figure S37. Representative flow cytometric analysis of MDSCs ($CD45^+CD11b^+Gr\text{-}1^+$) in the tumors.

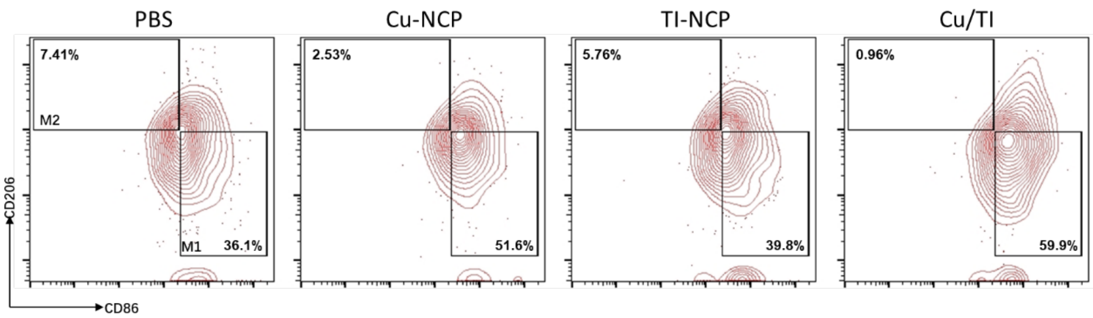

Figure S38. Representative flow cytometric analysis of M1(CD45$^+$CD11b$^+$F4/80$^+$CD86$^+$CD206$^-$) and M2(CD45$^+$CD11b$^+$F4/80$^+$CD86$^-$CD206$^+$) macrophage in tumors.

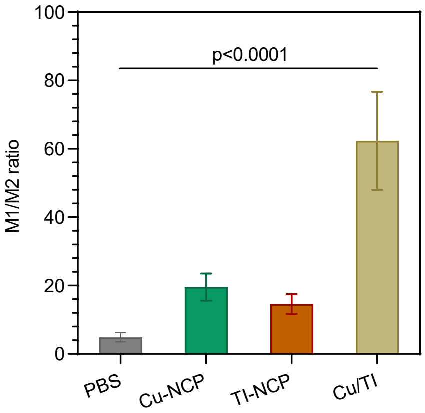

Figure S39. M1/M2 ratios in the tumors. Data are represented as mean ± SD, n = 3. One-way ANOVA with Tukey's correction for statistical significance.

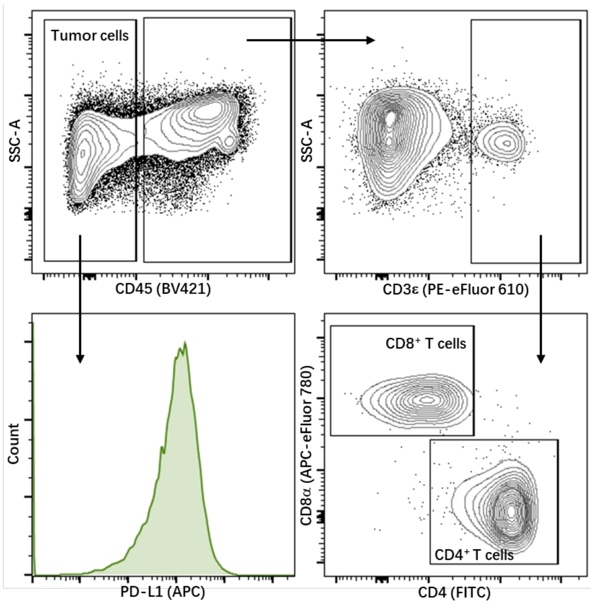

Figure S40. Gating strategies in single and live cells for CD8$^+$ T cells, CD4$^+$ T cells and PD-L1 expression of tumor cells in the tumors.

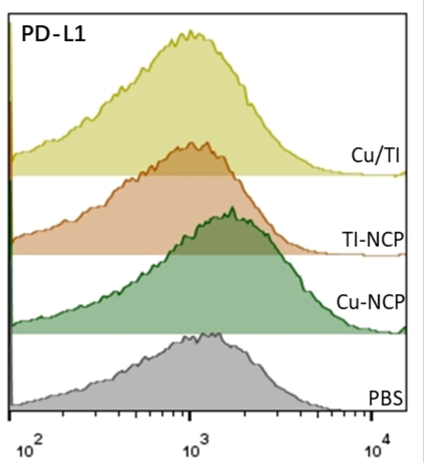

Figure S41. Representative flow cytometric analysis of PD-L1 expression of tumor cells *in vivo*.

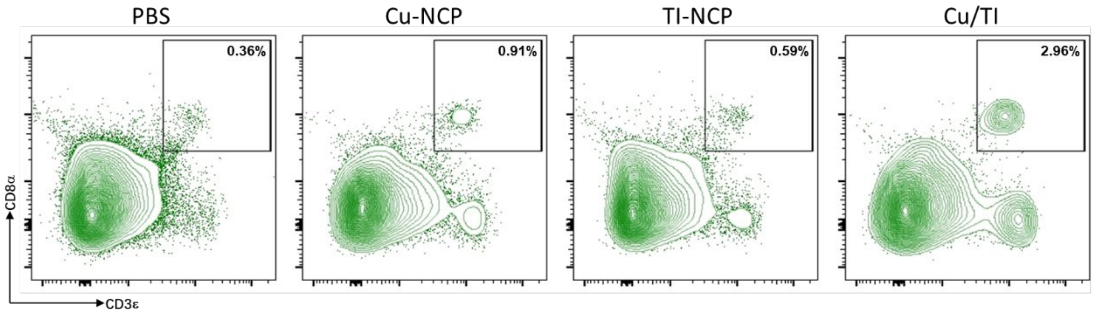

Figure S42. Representative flow cytometric analysis of $CD8^+$ T cells ($CD45^+CD3\varepsilon^+CD8\alpha^+$) in the tumors.

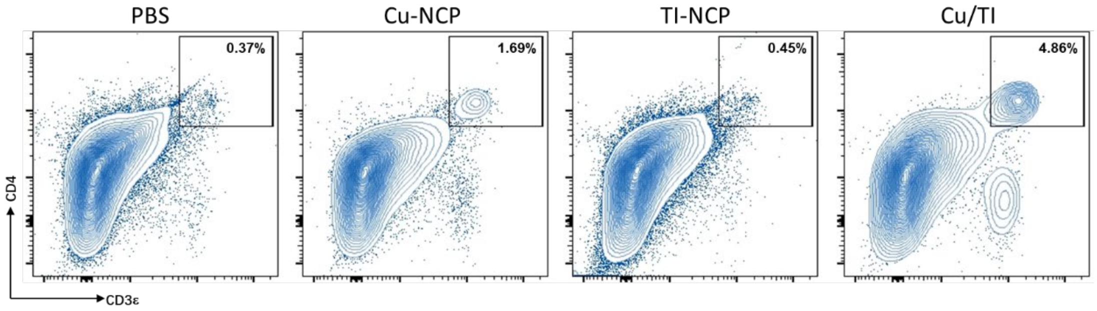

Figure S43. Representative flow cytometric analysis of $CD4^+$ T cells ($CD45^+CD3\varepsilon^+CD4^+$) in the tumors.

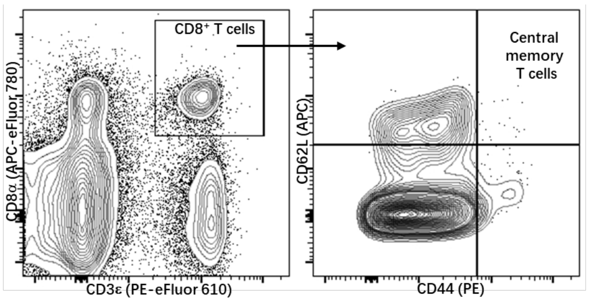

Figure S44. Gating strategies in single and live cells for central memory T cells in the spleens.

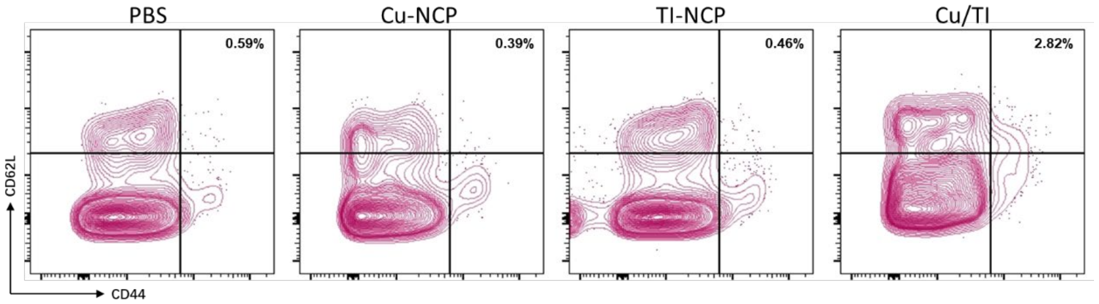

Figure S45. Representative flow cytometric analysis of central memory T cells ($CD3\varepsilon^{+}CD8\alpha^{+}$ $CD44^{+}CD62L^{+}$) in the spleens.

Table S1. IC_{50} values of Cu and IOX1 under different conditions in 4T1 cells. Data are represented as mean ± SD, *n* = 3.

	**$CuCl_2$**	**IOX1**	**IOX1:Cu=1:1**	**IOX1 in 1μM Cu**
IC_{50} (μM)	>100	>100	12.14 ± 0.78	14.36 ± 0.73

Table S2. IC_{50} values of Cu in different IOX1 concentrations in 4T1 cells. Data are represented as mean ± SD, *n* = 3.

**IOX1 (μM)**	**10**	**25**	**50**
Cu IC_{50} (μM)	40.93 ± 10.22	3.19 ± 0.87	2.28 ± 0.70

Table S3. IC_{50} values of Cu and TI under different conditions in 4T1 cells. Data are represented as mean ± SD, *n* = 3.

	**TI**	**TI in 1 μM Cu**	**Cu in 25 μM TI**
IC_{50} (μM)	>100	11.75 ± 2.38	2.54 ± 0.70

Table S4. Composition, hydrodynamic size and distribution, and zeta potential of different nanoparticles. Data are represented as mean ± SD, *n* = 3.

**Particle**	**Core**	**Shell**			**Size (nm)**	**PDI**	**Zeta potential (mV)**
	Cu<br>DOPA	TI	TPP-pyro	Cholesterol<br>DOPC<br>DSPE-PEG_{2000}			

Cu-bare	√				68.9 ± 1.2	0.198	
Cu-NCP	√			√	110.3 ± 2.1	0.229	0.45 ± 0.06
TI-NCP		√		√	141.2 ± 3.4	0.272	2.4 ± 0.6
Cu/TI	√	√		√	137.5 ± 2.4	0.237	1.3 ± 0.3
Cu/TPP-pyro	√		√	√	142.5 ± 2.9	0.233	1.7 ± 0.2

Table S5. Cytotoxicity of different nanoparticles in HEK293T cells. For Cu/TI the IC_{50} is based on concentration of Cu (with 25 equivalents of TI). Data are represented as mean ± SD, n = 3.

	**Cu-NCP**	**TI-NCP**	**Cu/TI**
IC_{50} (μM)	>100	>100	2.98 ± 0.29

Table S6. Cu pharmacokinetics of BALB/c mice following a single intravenous injection of Cu/TPP-pyro with one compartment model fitting. Data are represented as mean ± SD, n = 3.

**Parameters**	**Unit**	**Cu/TPP-pyro NCP**
C_0	μg/ml	4.93 ± 0.31
C_{min}	μg/ml	1.15 ± 0.56
$t_{1/2}$	hour	7.23 ± 1.63
k	$hour^{-1}$	0.096 ± 0.014
$AUC_{0\text{-}inf\_obs}$	μg/ml×hour	273.6 ± 26.3

Table S7. TPP-pyro pharmacokinetics of BALB/c mice following a single intravenous injection of Cu/TPP-pyro with one compartment model fitting. Data are represented as mean ± SD, n = 3.

**Parameters**	**Unit**	**Cu/TPP-pyro NCP**
C_0	$\times 10^8$[p/s/cm^2/sr]/[μW/cm^2]	12.16 ± 0.84
C_{min}	$\times 10^8$[p/s/cm^2/sr]/[μW/cm^2]	1.94 ± 0.25
$t_{1/2}$	hour	9.57 ± 2.21
k	$hour^{-1}$	0.072 ± 0.023
$AUC_{0\text{-}inf\_obs}$	$\times 10^8$[p/s/cm^2/sr]/[μW/cm^2] ×hour	237.1 ± 13.8

Table S8. Cytotoxicity of different nanoparticles in RAW 264.7 cells. For Cu/TI the IC_{50} is based on concentration of Cu (with 25 equivalents of TI). Data are represented as mean ± SD, n = 3.

	**Cu-NCP**	**TI-NCP**	**Cu/TI**
IC_{50} (μM)	>100	>100	8.78 ± 1.44

References

[1] Y. Li, W. Han, C. He, X. Jiang, Y. Fan, W. Lin, *Bioconjug Chem* **2021**, *32*, 2318-2326.

[2] Y. Li, J. Liu, Y. Chen, R. R. Weichselbaum, W. Lin, *Advanced Science* **2024**, *n/a*, 2310309.

[3] D. Guanghua, A. D. Guido, F. Werner, G. Christoph, H. Volker, K. Reiner, K. Alexandra, T. Laura, A. F. Anna, D. Günther, *Radiation Research* **2011**, *176*, 706-715.

[4] P. Tsvetkov, S. Coy, B. Petrova, M. Dreishpoon, A. Verma, M. Abdusamad, J. Rossen, L. Joesch-Cohen, R. Humeidi, R. D. Spangler, J. K. Eaton, E. Frenkel, M. Kocak, S. M. Corsello, S. Lutsenko, N. Kanarek, S. Santagata, T. R. Golub, *Science* **2022**, *375*, 1254-1261.

[5] B. Schmid, J. Schindelin, A. Cardona, M. Longair, M. Heisenberg, *BMC Bioinformatics* **2010**, *11*, 274.
